# A Kinase-Independent Function of CDK6 Links the Cell Cycle to Tumor Angiogenesis

**DOI:** 10.1016/j.ccr.2013.07.012

**Published:** 2013-08-12

**Authors:** Karoline Kollmann, Gerwin Heller, Christine Schneckenleithner, Wolfgang Warsch, Ruth Scheicher, Rene G. Ott, Markus Schäfer, Sabine Fajmann, Michaela Schlederer, Ana-Iris Schiefer, Ursula Reichart, Matthias Mayerhofer, Christoph Hoeller, Sabine Zöchbauer-Müller, Dontscho Kerjaschki, Christoph Bock, Lukas Kenner, Gerald Hoefler, Michael Freissmuth, Anthony R. Green, Richard Moriggl, Meinrad Busslinger, Marcos Malumbres, Veronika Sexl

**Affiliations:** 1 Institute of Pharmacology and Toxicology, University of Veterinary Medicine Vienna, 1210 Vienna, Austria; 2 Institute of Animal Breeding and Genetics, University of Veterinary Medicine Vienna, 1210 Vienna, Austria; 3 Clinical Division of Oncology, Department of Medicine I, Comprehensive Cancer Center, Medical University of Vienna, 1090 Vienna, Austria; 4 Institute of Pharmacology, Center of Biomolecular Medicine and Pharmacology, Medical University of Vienna, 1090 Vienna, Austria; 5 Department of Clinical Pathology, Medical University of Vienna, 1090 Vienna, Austria; 6 Department of Laboratory Medicine, Medical University of Vienna, 1090 Vienna, Austria; 7 Department of Dermatology, Medical University of Vienna, 1090 Vienna, Austria; 8 Research Institute of Molecular Pathology, Vienna Biocenter, 1030 Vienna, Austria; 9 CeMM Research Center for Molecular Medicine of the Austrian Academy of Sciences, 1090 Vienna, Austria; 10 Ludwig Boltzmann Institute for Cancer Research, 1090 Vienna, Austria; 11 Department of Pathology, Medical University of Graz, 8036 Graz, Austria; 12 Cambridge Institute for Medical Research and Wellcome Trust/MRC Stem Cell Institute, University of Cambridge, Cambridge CB2 0XY, UK; 13 Department of Hematology, University of Cambridge, Cambridge CB2 0XY, UK; 14 Department of Hematology, Addenbrooke’s Hospital, Cambridge CB2 0XY, UK; 15 Cell Division and Cancer Group, Molecular Oncology Programme, Centro Nacional de Investigaciones Oncológicas (CNIO), 28029 Madrid, Spain

## Abstract

In contrast to its close homolog CDK4, the cell cycle kinase CDK6 is expressed at high levels in lymphoid malignancies. In a model for *p185*^*BCR-ABL+*^ B-acute lymphoid leukemia, we show that CDK6 is part of a transcription complex that induces the expression of the tumor suppressor p16^INK4a^ and the pro-angiogenic factor VEGF-A. This function is independent of CDK6’s kinase activity. High CDK6 expression thus suppresses proliferation by upregulating p16^INK4a^, providing an internal safeguard. However, in the absence of p16^INK4a^, CDK6 can exert its full tumor-promoting function by enhancing proliferation and stimulating angiogenesis. The finding that CDK6 connects cell-cycle progression to angiogenesis confirms CDK6’s central role in hematopoietic malignancies and could underlie the selection pressure to upregulate CDK6 and silence p16^INK4a^.

## Graphical Abstract

**Figure:**
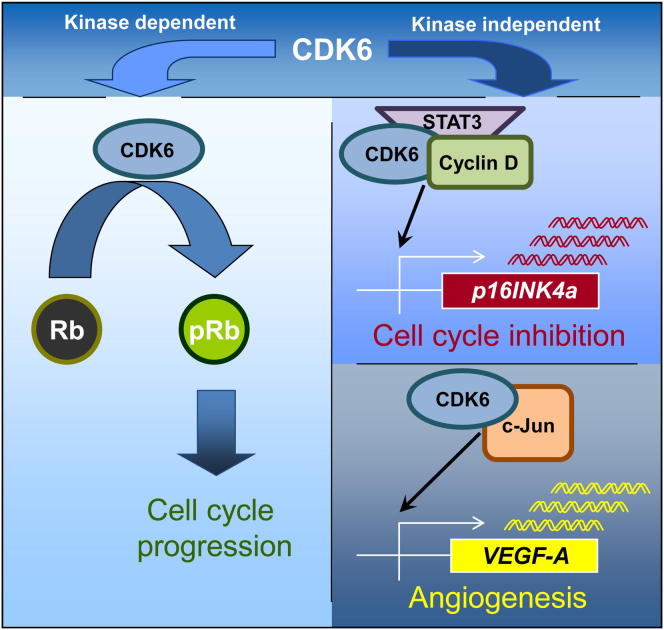

## Significance

**Aberrant growth control is one hallmark of cancer cells. The cyclin-dependent kinase CDK6 promotes cell-cycle progression and is expressed at high levels in lymphoid tumors. We present evidence for a function of CDK6 as a transcriptional regulator that is unrelated to its kinase activity. Part of the CDK6-dependent gene signature is *VEGF-A*, which promotes tumor angiogenesis and controls de novo formation of blood vessels. CDK6 thus represents a factor that regulates tumor growth while also ensuring the supply of oxygen and energy to the tumor. Available anticancer drugs targeting CDK6 focus on CDK6’s kinase-dependent functions. Our insights may reshape future strategies to develop CDK6 inhibitors, allowing the simultaneous inhibition of cell-cycle progression and CDK6’s other, kinase-independent functions.**

## Introduction

Cell cycle deregulation is a common feature of human cancer (Cordon-Cardo, 1995, Deshpande et al., 2005, Kim and Diehl, 2009, Malumbres and Barbacid, 2001). Accordingly, cell-cycle kinases represent promising targets for the development of low-molecular-weight compounds for use in cancer therapy (Cicenas and Valius, 2011, Malumbres, 2012). Cyclin dependent kinases (Cdks) belong to the core cell-cycle machinery and comprise a family of serine/threonine kinases that exert their catalytic activity when bound to their partners, the cyclins (Sherr and Roberts, 1999). The close homologs CDK4 and CDK6 are ubiquitously expressed. They bind D-type cyclins and are then able to phosphorylate the retinoblastoma protein to relieve the transcriptional repression of E2F-dependent genes, thereby driving cells through the G1 phase into the S phase of the cell cycle (Classon and Harlow, 2002, Ekholm and Reed, 2000, Matsushime et al., 1994). Experiments with gene-deficient mice have facilitated the elucidation of partially overlapping and redundant physiologic roles for CDK4 and CDK6. Apart from minor tissue-specific abnormalities, *Cdk4* and *Cdk6* single knockout mice are viable and fertile (Malumbres et al., 2004, Zou et al., 2002). The deletion of both *Cdk4* and *Cdk6* induces late embryonic lethality due to defects in hematopoiesis (Kozar and Sicinski, 2005, Malumbres et al., 2004).

Cell-cycle inhibitors block cell-cycle progression by binding and inhibiting cell-cycle kinases. The CDK4- and CDK6-cyclin D complexes are subject to inhibition by p21^cip^ and p27^kip^. In addition, the INK4 family members (p16^INK4a^, p15^INK4b^, p18^INK4c^, p19^INK4d^) inhibit monomeric CDK4 and CDK6 and thus prevent complex formation with cognate cyclins (Cánepa et al., 2007, Pavletich, 1999, Sherr and Roberts, 1999, Vidal and Koff, 2000).

Cell-cycle components are frequently altered or mutated in human cancer, reflecting the deregulated growth of transformed cells. Mutations in exon 2 of *CDK4* have been related to hereditary melanoma (Bressac-de-Paillerets et al., 2002, Goldstein et al., 2006, Wölfel et al., 1995). Recently, two novel mutations in the N-terminal domain of *CDK4* were related to head and neck cancer (Sabir et al., 2012). CDK4 has also been characterized as an essential component for *c-neu/ERBB-2*-induced breast cancer (Landis et al., 2006). *Cdk4*^−*/*−^ breast tissue is indistinguishable from the normal mouse mammary gland; however, *c-neu/ERBB-2-*dependent tumor development is completely abrogated in the absence of CDK4 (Landis et al., 2006, Malumbres and Barbacid, 2006, Reddy et al., 2005). No mutations in human *CDK6* have been identified, although enhanced CDK6 expression has been documented in lymphoma and leukemia (Chilosi et al., 1998, Lien et al., 2000, Nagel et al., 2008, Schwartz et al., 2006), and several reports have documented chromosomal translocations in patients suffering from B-lymphoid malignancies involving *CDK6*. In these patients, the aberrant and increased expression of CDK6 has been proposed to be the cause and/or driving force for the disease (Brito-Babapulle et al., 2002, Chen et al., 2009, Hayette et al., 2003, Parker et al., 2012). In addition, recent papers describe altered microRNA regulation (i.e., miR-124a, miR-29) resulting in an upregulation of CDK6 in lymphoid malignancies (Agirre et al., 2009, Rodriguez-Otero et al., 2011, Wong et al., 2011, Zhao et al., 2010). Nevertheless, there is still no formal proof that CDK6 has a role in initiating B-lymphoid leukemia or in driving the expansion of a leukemic clone.

CDK6 has also been implicated in thymic lymphoma formation in a transgenic mouse model in which lymphoma development is triggered by a constitutively active AKT. In this particular setting, lymphomas did not develop in the absence of CDK6 (Hu et al., 2009). We have shown that loss of the AP-1 transcription factor JUNB—as frequently observed in human leukemia (Mao et al., 2003, Yang et al., 2003) - is associated with more aggressive disease and a significantly accelerated progression of p185^BCR-ABL^-induced leukemia (Szremska et al., 2003). The expression of CDK6 is consistently elevated in the highly malignant *JunB* deficient cells (Ott et al., 2007). In line with this, the lack of c-JUN, an antagonist of JUNB, leads to the downregulation of CDK6 and as a result to reduced proliferation of p185^BCR-ABL^-transformed cells as well as to prolonged disease onset. The data were confirmed by using the *Cdk6*^−*/*−^ mouse in a *p185*^*BCR-ABL*^ leukemia model (Kollmann et al., 2011a). Together with the reports of elevated CDK6 protein levels in human disease, these findings prompted us to investigate the role of CDK6 in lymphoid malignancies.

## Results

### Forced CDK6 Expression Suppresses Tumor Formation

**Figure 1:**
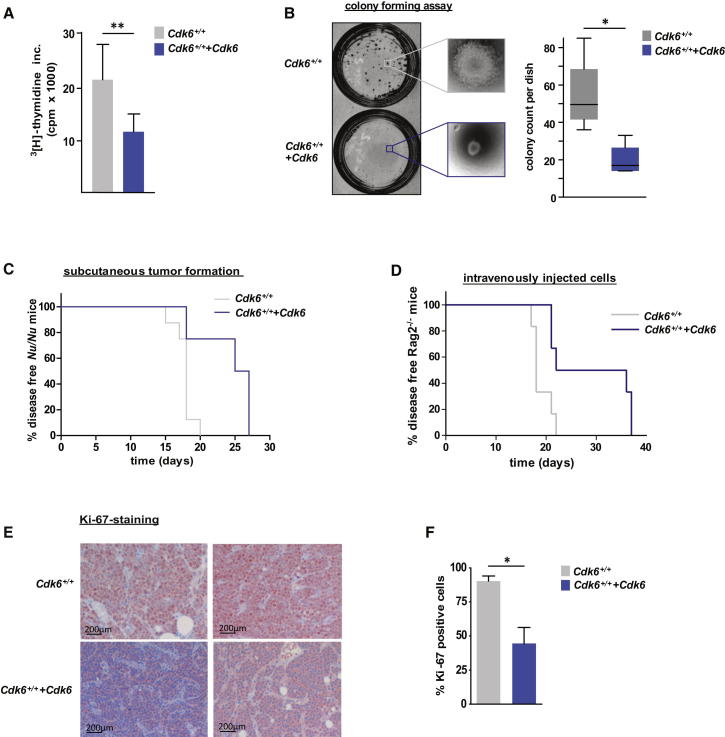
Enforced Expression of CDK6 in p185^BCR-ABL^-Transformed Cells Unmasks Its Tumor-Suppressing Activity (A) ^3^[H]-thymidine incorporation of *Cdk6*^*+/+*^ (expressing a pMSCV-puro-based retrovirus) and *Cdk6*^*+/+*^*+Cdk6* (expressing a pMSCV-Cdk6-puro-based retrovirus) p185^BCR-ABL^-transformed cells (n = 4; ^∗∗^p = 0.009). (B) Colony-forming assays were performed by seeding a defined number of p185^BCR-ABL^-transformed *Cdk6*^*+/+*^ or *Cdk6*^*+/+*^*+Cdk6* cells in growth factor-free methylcellulose. Left side: representative set of pictures. Right side: number of colonies per dish after incubation for 5 days (n = 4/genotype; ^∗^p = 0.023). (C) Kaplan-Meier plot of *Nu/Nu* mice injected subcutaneously with *Cdk6*^*+/+*^ or *Cdk6*^*+/+*^*+Cdk6* cells (n = 4 cell lines/genotype; n = 2 mice/cell line; mean survival: 18 [*Cdk6*^*+/+*^] versus 26 [*Cdk6*^*+/+*^*+Cdk6*] days; ^∗∗^p = 0.002). (D) Kaplan-Meier plot of *Rag2*^−*/*−^ mice transplanted intravenously with *Cdk6*^*+/+*^ or *Cdk6*^*+/+*^*+Cdk6* cells (n = 3 cell lines/genotype; n = 6 mice/genotype; mean survival: 18 [*Cdk6*^*+/+*^] and 29 [*Cdk6*^*+/+*^*+Cdk6*] days; ^∗^p = 0.014). (E and F) Immunohistochemical stainings for the proliferation marker Ki-67 of *Cdk6*^*+/+*^ (n = 4) and *Cdk6*^*+/+*^*+Cdk6* (n = 9) tumors were quantified with HistoQuest software. A representative set of pictures is given (E). Original magnification 20×. Bar graphs depict percentage of Ki-67-positive tumor cells (F; p = 0.029 [^∗^]). Error bars indicate the mean ± SEM. See also Figure S1.

To investigate the consequences of increasing CDK6 expression in B-lymphoid leukemia/lymphoma, we generated stable p185^BCR-ABL^-transformed pro B cell lines. These were infected with either a pMSCV-puro (*Cdk6*^*+/+*^) or a pMSCV-Cdk6-puro based retrovirus (*Cdk6^+/+^+Cdk6*). Contrary to our expectations, *Cdk6^+/+^+Cdk6* cells displayed a strong reduction of proliferation (confirmed by three different experimental techniques, see Figure 1A; Figures S1A–S1C available online). Moreover, cells with high CDK6 levels formed fewer and smaller colonies in growth factor-free methylcellulose (Figure 1B). We did not find any increase in apoptosis or any signs of senescence on inducing CDK6 expression (Figures S1D–S1F and data not shown). No major changes in subcellular localization of CDK6 were observed upon enforced expression (Figure S1G). Consistently with their in vitro phenotype, *Cdk6^+/+^+Cdk6* cells gave rise to subcutaneous lymphoma-like tumors or leukemia with increased latency (Figures 1C and 1D). We confirmed the reduced proliferation of *Cdk6^+/+^+Cdk6* cells in vivo by staining subcutaneous tumor sections for the proliferation marker Ki-67 (Figures 1E and 1F). These experiments are in line with a tumor-suppressing function of CDK6.

### CDK6 Acts as a Transcriptional Regulator on the *p16*^*INK4a*^ Promoter

**Figure 2:**
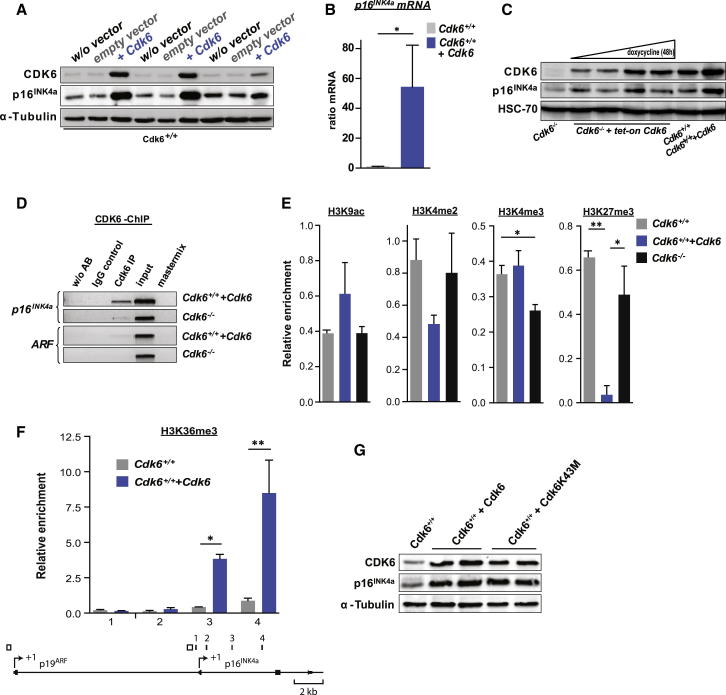
CDK6 Acts as a Transcriptional Regulator of p16^INK4a^ (A) Immunoblot for CDK6 and p16^INK4a^ of three individually derived *Cdk6*^*+/+*^ cell lines with (+Cdk6) or without (w/o vector, empty vector) enforced CDK6 expression. (B) *p16*^*INK4a*^ mRNA levels of *Cdk6*^*+/+*^ and *Cdk6*^*+/+*^*+Cdk6* cells were analyzed with qPCR (n = 6; ^∗^p = 0.03). (C) Immunoblot for CDK6 and p16^INK4a^ of *Cdk6*^−*/*−^ cells expressing a doxycycline-inducible *tet-on Cdk6* vector (*Cdk6*^−*/*−^*+tet-on Cdk6*). Lane 1: *Cdk6*^−*/*−^ cells. Lanes 2–5: *Cdk6*^−*/*−^*+tet-on Cdk6* cells 48 hr after treatment with doxycycline (0, 0.3, 1, 3 μM). Lane 6: *Cdk6*^*+/+*^ cells. Lane 7: *Cdk6*^*+/+*^*+Cdk6* cells. (D) ChIP assays were performed using *Cdk6*^*+/+*^*+Cdk6* and *Cdk6*^−*/*−^ cells. Protein-DNA complexes were immunoprecipitated using an anti-CDK6-antibody and analyzed by PCR for the presence of *p16*^*INK4a*^ and as specificity control of *ARF* promoter sequence (region depicted in Figure 2F as open rectangles). (E) Promoter ChIP assays were performed using *Cdk6*^+/+^, *Cdk6*^*+/+*^*+Cdk6*, and *Cdk6*^−*/*−^ cells. Protein-DNA complexes were immunoprecipitated using antibodies specific for the indicated histone modification. ChIP DNA was analyzed with qPCR for the presence of a *p16*^*INK4a*^ promoter sequence (region 1 in Figure 2F). The relative enrichment to a control region is shown (*Tbp* promoter for H3K9ac, H3K4me2, and H3K4me3; *Neurog1* promoter for H3K27me3). The mean and SEM of two independent experiments is shown (H3K4me3: *Cdk6*^+/+^ versus *Cdk6*^−*/*−^, ^∗^p < 0.05; H3K27me3: *Cdk6*^+/+^ versus *Cdk6*^*+/+*^*+Cdk6,*^∗∗^p < 0.01; *Cdk6*^*+/+*^*+Cdk6* versus *Cdk6*^−*/*−^, ^∗^p < 0.05). (F) H3K36me3 ChIP assays were performed using *Cdk6*^+/+^ and *Cdk6*^*+/+*^*+Cdk6* cells. Protein-DNA complexes were immunoprecipitated using an antibody specific for H3K36me3. ChIP DNA was analyzed with qPCR for the presence of *p16*^*INK4a*^ sequences 1–4, which are depicted as filled black rectangles in the lower panel (middle of the amplicon relative to the TSS [arrow symbol marked +1] of *p16*^*INK4a*^: 1, *−*169 base pairs; 2, *+*538 base pairs; 3, +2,278 base pairs; 4, +4,312 base pairs). The relative enrichment to a *Gapdh* gene body region is shown. The mean and SEM of two independent experiments is shown (sequence 3: *Cdk6*^+/+^ versus *Cdk6*^*+/+*^*+Cdk6,*^∗^p < 0.05; sequence 4: *Cdk6*^+/+^ versus *Cdk6*^*+/+*^*+Cdk6,*^∗∗^p < 0.01). (G) Immunoblot for CDK6 and p16^INK4a^ of *Cdk6*^+/+^, *Cdk6*^*+/+*^*+Cdk6*, and *Cdk6*^*+/+*^*+Cdk6K43M* cells. See also Figure S2.

To investigate the underlying mechanism, we examined the expression of several genes known to be important for cell-cycle control. Elevated CDK6 expression was consistently accompanied by high levels of the cell-cycle inhibitor and tumor suppressor p16^INK4a^, while protein levels of other members of the INK4 family (p15^INK4b^, p18^INK4c^, p19^INK4d^), p19^ARF^ as well as p21^CIP1^ and p27^KIP1^ remained unchanged or were hardly expressed at all (Figure 2A; Figures S2A and S2B). These findings were recapitulated in murine embryonic fibroblasts (MEFs) that had not yet undergone senescence (Figure S2C). The upregulation of p16^INK4a^ is not caused by increased protein stability as it persisted upon treatment with the proteasome inhibitor bortezomib (Figure S2D). In contrast to CDK6, high expression of CDK4 in p185^BCR-ABL+^ leukemic cells did not increase p16^INK4a^ expression, nor was any change in tumor growth observed (Figures S2E–S2H).

Enforced expression of CDK6 led to a pronounced increase in the levels of *p16*^*INK4a*^ mRNA and pre-mRNA (Figure 2B; Figure S2I), indicating a role for CDK6 in regulating the transcription of p16^INK4a^. In line with this, expression of CDK6 in p185^BCR-ABL^-transformed *Cdk6*^−*/*−^ cell lines resulted in a p16^INK4a^ upregulation as well as a decreased proliferation (Figures S2J–S2L). The generation of p185^BCR-ABL^-transformed *Cdk6*^−*/*−^ cells expressing a doxycycline-inducible CDK6 construct showed that the induction of p16^INK4a^ occurs at concentrations that are within a physiologic range (Figure 2C). Chromatin immunoprecipitation (ChIP) experiments revealed that CDK6 binds specifically to the *p16*^*INK4a*^ promoter, while no binding to the *p19*^*ARF*^ promoter was detected (Figure 2D; Figure S6I). These findings define CDK6 as part of a transcriptional complex that regulates the tumor suppressor p16^INK4a^. In line with this finding, we detected the loss of the inhibitory histone mark H3K27me3, which controls p16^INK4a^ expression (Figure 2E; Agger et al., 2009, Barradas et al., 2009). Consistently, the level of H3K36me3 is significantly increased: this marker parallels transcriptional activity (Figure 2F).

To investigate whether CDK6 exerts its action on the promoter by phosphorylation of a hitherto unknown substrate, we tested whether the kinase-inactivated CDK6 mutant CDK6K43M (Zacharek et al., 2005) could upregulate p16^INK4a^. p185^BCR-ABL^-transformed *Cdk6*^−*/*−^ and wild-type (WT) cells were infected with a pMSCV-puro-based retrovirus containing *Cdk6K43M* to generate stable cell lines (Figure 2G; Figure S2J). Surprisingly, upregulation of *p16*^*INK4a*^ mRNA and protein is kinase-independent (Figure 2G; Figures S2M, S2J, and S2K) and expression of the mutant CDK6K43M reduces cell growth similarly to expression of WT CDK6 (Figures S2N and S2L). In line with these findings, we detected the CDK6K43M mutant at the *p16*^*INK4a*^ promoter (Figure S6I). These experiments indicate the existence of a kinase-independent function of CDK6 as a transcriptional regulator of the tumor suppressor p16^INK4a^.

### Inverse Protein Expression Levels of CDK6 and p16^INK4a^ in Human Tumor Tissue

**Figure 3:**
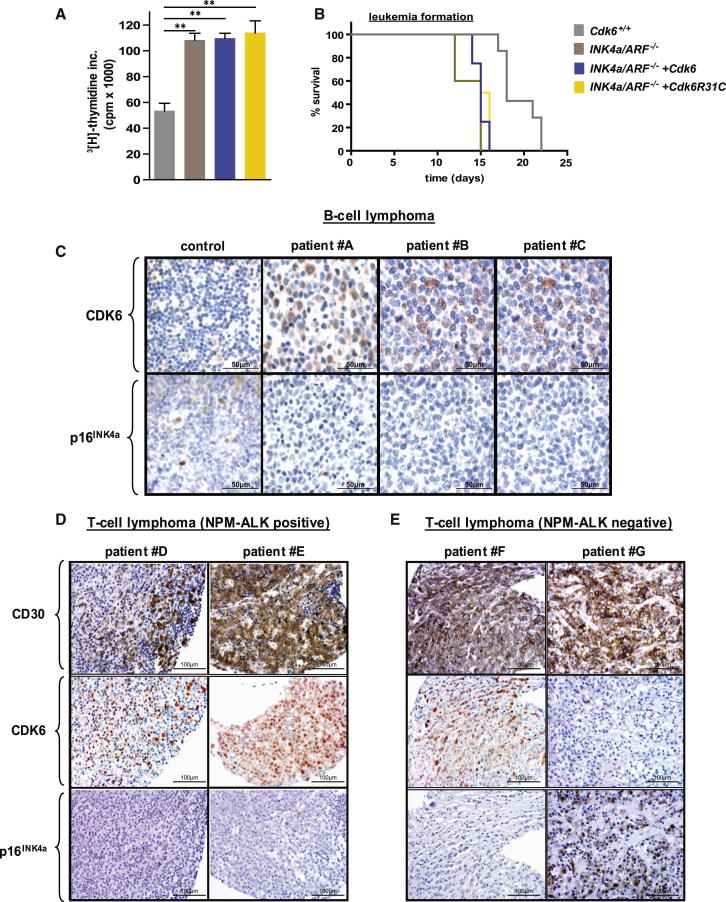
Inverse Relation between CDK6 and p16^INK4a^ Expression in Human Lymphomas (A) [^3^H]thymidine incorporation of *Cdk6*^*+/+*^, *INK4a/ARF*^−*/*−^*, INK4a/ARF*^−/−^*+Cdk6*, and *INK4a/ARF*^−*/*−^*+Cdk6R31C* p185^BCR-ABL^-transformed cells (n = 3; *Cdk6*^*+/+*^ versus *INK4a/ARF*^−*/*−^, ^∗∗^p = 0.002; *INK4a/ARF*^−*/*−^*+Cdk6*, ^∗∗^p = 0.001; *INK4a/ARF*^−*/*−^*+Cdk6R31C,*^∗∗^p = 0.005). (B) Kaplan-Meier plot of *Rag2*^−*/*−^ mice intravenously transplanted with *Cdk6*^*+/+*^, *INK4a/ARF*^−*/*−^*, INK4a/ARF*^−/−^*+Cdk6*, and *INK4a/ARF*^−*/*−^*+Cdk6R31C* cells (n = 3 cell lines/genotype; mean survival: 18 [*Cdk6*^*+/+*^], 15 [*INK4a/ARF*^−*/*−^], 15 [*INK4a/ARF*^−*/*−^*+Cdk6*], and 15.5 [*INK4a/ARF*^−*/*−^*+Cdk6R31C*] days; ^∗^p = 0.02). (C) Immunohistochemical stainings of a B cell lymphoma tissue array including 16 lymphoma samples and two control lymph nodes for CDK6 and p16^INK4a^. Representative examples including different types of B cell lymphoma, a diffuse large B cell lymphoma (patient A), two follicular lymphomas (patients B and C), and one control lymph node (left) are depicted. Original magnification 20×. (D and E) The expression of CD30, CDK6, and p16^INK4a^ was analyzed for 17 *NPM-ALK*-positive (E) and 11 *NPM-ALK*-negative (D) lymphoma cases with immunohistochemistry. Representative cases for *NPM-ALK*-positive (patients D and E) and *NPM-ALK*-negative (patients F and G) cases are depicted. Original magnification 20×. Error bars indicate the mean ± SEM. See also Figure S3.

To examine whether p16^INK4a^ expression accounts for the growth inhibition caused by CDK6, we expressed CDK6 in p185^BCR-ABL^-transformed cells deficient for *p16*^*INK4a*^*/p19*^*ARF*^ (*INK4a/ARF*^−*/*−^). Those cells showed no growth inhibitory effects or reduction of leukemogenesis upon enforced CDK6 expression (Figures 3A and 3B). To substantiate the role of p16^INK4a^ in a negative feedback loop counteracting high levels of CDK6 expression, we used the CDK6R31C mutant, which is incapable of binding INK4 proteins (Grossel et al., 1999). Enforced expression of this mutant should provoke p16^INK4a^ expression but fail to induce a growth inhibitory effect based on the inability to bind p16^INK4a^. Indeed, the reconstitution of p185^BCR-ABL^-transformed *Cdk6*^−*/*−^ and WT cells with a pMSCV-puro-based retrovirus containing *Cdk6R31C* did not mimic the growth inhibitory effect of CDK6 despite the presence of high levels of p16^INK4a^ (Figures S2K–S2P). These experiments confirm that the tumor-suppressing effect of high CDK6 expression results from a negative feedback loop mediated by p16^INK4a^. Accordingly, in the presence of an intact p16^INK4a^ regulation, there is only a narrow window for CDK6 acting as growth promoter.

Our model makes a testable prediction: high CDK6 expression in a lymphoid tumor should confer a growth advantage only when expression of p16^INK4a^ is disrupted. Support for the prediction comes from an analysis of patient samples. Levels of CDK6 and p16^INK4a^ protein were analyzed in two human tissue arrays, one for B cell malignancies, and one for T cell malignancies. Figure 3C compiles three examples of a tissue array consisting of 16 different cases of B cell lymphoma as well as a control lymph node. In all cases, we found high levels of CDK6 expression accompanied by reduced or undetectable expression of p16^INK4a^. The tissue array for T cell malignancies consisted of 28 cases of anaplastic large cell lymphoma (ALCL), among which 17 carried the NPM-ALK fusion kinase (Figures 3D and 3E). Because ALCL cells diffusely infiltrate affected lymph nodes, we used CD30 staining to define the malignant cells. We found an inverse relationship between CDK6 and p16^INK4a^ in all samples (Chi-square test, χ^2^ = 11.603, p = ∼0.02). The majority of cases had high CDK6 expression and no or scarcely detectable expression of p16^INK4a^. However, 4 of 11 NPM-ALK-negative tumors displayed the opposite phenotype: they had high p16^INK4a^ levels but lacked immunoreactivity against CDK6 antibodies (Figure 3E). The correlation was verified by HistoQuest-assisted analysis (Figures S3A and S3B). The array experiments revealed an inverse relationship between p16^INK4a^ and CDK6 expression in human B- and T-cell lymphoma cells, which was confirmed with immunofluorescence staining (Figure S3C).

### The CDK6-p16^INK4a^ Loop in Murine NPM-ALK-Driven T-Lymphoid Disease

**Figure 4:**
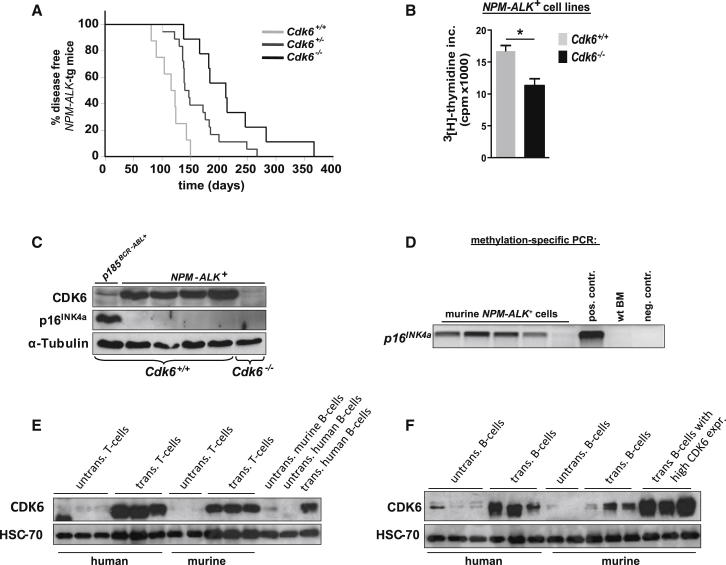
CDK6 Regulates NPM-ALK-Induced Disease Progression (A) *Cdk6*^*+/+*^, *Cdk6*^*+/*−^, and *Cdk6*^−*/*−^ mice crossed with *NPM-ALK* transgenic (tg) mice developed a T cell lymphoma after several weeks (n ≥ 8; mean survival 120 [*Cdk6*^*+/+*^], 143.5 [*Cdk6*^*+/*−^], and 212 [*Cdk6*^−*/*−^] days; ^∗∗^p = 0.001). (B) [^3^H]thymidine incorporation of NPM-ALK-transformed *Cdk6*^*+/+*^ and *Cdk6*^−*/*−^ cells (n ≥ 3; ^∗^p = 0.009). (C) Immunoblot for CDK6 and p16^INK4a^ of NPM-ALK-transformed *Cdk6*^*+/+*^ and *Cdk6*^−*/*−^ cells. (D) Methylation-specific PCR of a part of the *p16*^*INK4a*^ promoter CpG island in NPM-ALK-transformed murine cells. The visible PCR product indicates the presence of methylated alleles. wt BM, bone marrow of a healthy mouse; pos. contr., control for methylated samples; neg. contr., control for unmethylated samples. (E) Immunoblot for CDK6 of untransformed human T cells (three individually derived samples), human T-lymphoid leukemic cell lines (Sudhl1, CCRF, PEER), untransformed murine T cells (two individually derived samples), murine *Cdk6*^+/+^ NPM-ALK-transformed cells, untransformed murine B cells, untransformed human B cells, and human B-lymphoid leukemic cell lines (SUP-B15). (F) Immunoblot for CDK6 of untransformed human B cells (three individually derived samples), human B-lymphoid leukemic cell lines (SUP-B15, RL-7, JVM2), untransformed murine B cells (two individually derived samples), murine *Cdk6*^+/+^ p185^BCR-ABL^-transformed cells (three individually derived cell lines), and murine *Cdk6*^+/+^*+Cdk6* p185^BCR-ABL^-transformed cells (three individually derived cell lines). Error bars indicate the mean ± SEM. See also Figure S4.

In conjunction with a previous report of AKT-induced thymoma formation (Hu et al., 2009), our human data indicate a role for CDK6 not only in B-lymphoid disease, but also in T-lymphoid tumor formation. In line with the expression data in human *NPM-ALK*^*+*^ samples, which indicate a key role for CDK6 in this disease entity, *NPM-ALK*-transgenic mice lacking *Cdk6* developed disease with a significantly prolonged latency and *NPM-ALK*^*+*^ *Cdk6*^−*/*−^ cells showed a reduced proliferation in vitro (Figures 4A and 4B). In cell lines derived from *NPM-ALK*^*+*^ *Cdk6*^*+/+*^ lymphomas, we found pronounced high CDK6 protein levels. As predicted by our model, this was accompanied by the loss of p16^INK4a^ protein (Figure 4C). The *p16*^*INK4a*^ promoter CpG island was methylated in four of five murine NPM-ALK^+^ lymphoma-derived cell lines, indicating that methylation is responsible for the loss of p16^INK4a^ (Figure 4D). To assess whether the level of CDK6-enforced expression in transformed murine cells is comparable to that observed in human patients, we analyzed levels of CDK6 protein in human and murine lymphoid cell lines side by side using antibodies that recognize conserved epitopes of murine and human CDK6. We found that levels of CDK6 were within the same range in mice and men (Figures 4E and 4F; Figures S4A–S4D). In both species, a significant upregulation of CDK6 protein occurs upon transformation of lymphoid cells as evident by a side-by-side comparison of transformed versus nontransformed cells (Figures 4E and 4F). These data led us to conclude that our model system reflects the situation in human patients.

### Correlation of CDK6 Expression and Tumor Angiogenesis

**Figure 5:**
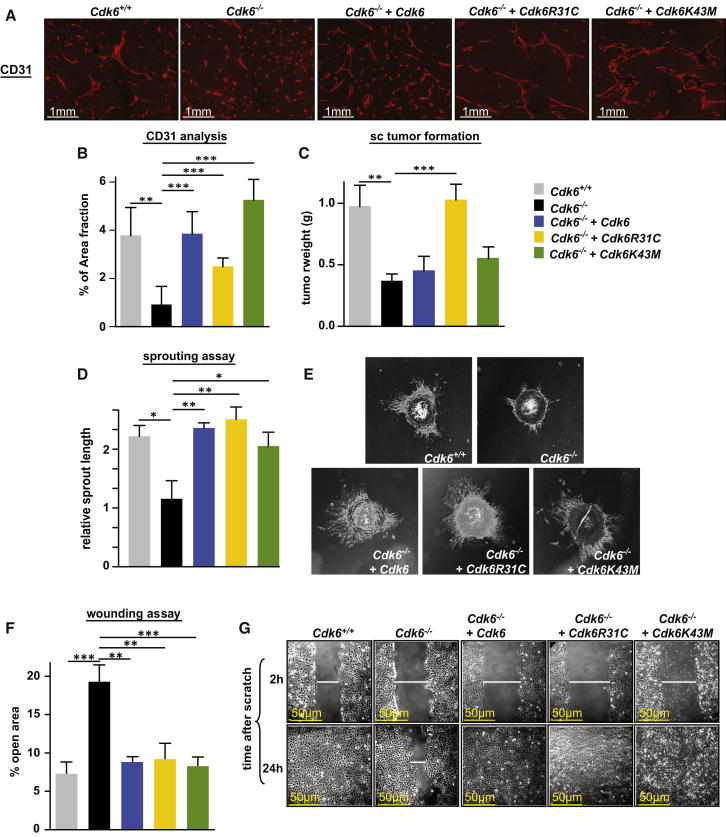
CDK6 Regulates Tumor Angiogenesis (A–C) *Cdk6*^*+/+*^*, Cdk6*^−*/*−^*, Cdk6*^−*/*−^*+Cdk6, Cdk6*^−*/*−^*+Cdk6R31C*, or *Cdk6*^−*/*−^*+Cdk6K43M* p185^BCR-ABL^-transformed cells were injected subcutaneously (sc) into *Nu/Nu* mice (n = 3 cell lines/genotype; n ≥ 6 tumors/genotype). (A) Immunofluorescence staining for CD31 (red) was performed to analyze blood vessel formation in sc tumors. Original magnification 20×. Representative cases of each genotype are depicted. (B) Quantitative assessment (HistoQuest) of the blood vessels of the subcutaneous tumors (n ≥ 4 tumors of three independent cell lines; *Cdk6*^−*/*−^ versus: *Cdk6*^*+/+*^, ^∗∗^p = 0.001; *Cdk6*^−*/*−^*+Cdk6*, ^∗∗∗^p < 0.0001; *Cdk6*^−*/*−^*+Cdk6R31C*, ^∗∗∗^p = 0.0002; *Cdk6*^−*/*−^*+Cdk6 K43M*, ^∗∗∗^p < 0.0001). (C) Tumor weight was detected after 8 days of injection (*Cdk6*^−*/*−^ versus: *Cdk6*^*+/+*^, ^∗∗^p = 0.003; *Cdk6*^−*/*−^*+Cdk6R31C*, ^∗∗∗^p = 0.0001). (D and E) Murine endothelial cell (mEC) spheroids were cultured in methylcellulose with 20% supernatant derived from indicated cells for 24 hr. (D) Quantitative analysis of the relative sprout length was measured with ImageJ software (n ≥ 3; *Cdk6*^−*/*−^ versus: *Cdk6*^*+/+*^, ^∗^p = 0.042; *Cdk6*^−*/*−^*+Cdk6*, ^∗∗^p = 0.002; *Cdk6*^−*/*−^*+Cdk6R31C*, ^∗∗^p = 0.009; *Cdk6*^−*/*−^*+Cdk6K43M*, ^∗^p = 0.038). (E) One representative set of pictures is given. (F and G) A monolayer wounding assay was performed to analyze migration of mECs incubated with supernatant derived from indicated cells. After 2 and 24 hr, pictures were taken and mEC migration quantified (% open area after 24 hr) with TScratch Software. (F) n ≥ 5; *Cdk6*^−*/*−^ versus: *Cdk6*^*+/+*^, ^∗∗∗^p = 0.0007; *Cdk6*^−*/*−^*+Cdk6*, ^∗∗^p = 0.0014; *Cdk6*^−*/*−^*+Cdk6R31C*, ^∗∗^p = 0.0092; *Cdk6*^−*/*−^*+Cdk6K43M*, ^∗∗∗^p = 0.0009). (G) One representative set of pictures is given. Error bars indicate the mean ± SEM. See also Figure S5.

Staining 13 human ALCL (11 *NPM-ALK*^*+*^ and 2 *NPM-ALK*^−^) samples for the angiogenic marker CD31 indicated an increased density of blood vessels in tumors expressing high levels of CDK6 (Figures S5A and S5B). Additionally, we investigated 33 diffuse large B cell lymphoma (DLBCL) cases for the correlation of CDK6 expression and blood vessel density (Figures S5C and S5D). The analysis of the DLBCL samples clearly supports the notion that CDK6 promotes angiogenesis in hematopoietic malignancies. To investigate whether CDK6 directly regulates angiogenesis, we reconstituted *Cdk6*-deficient p185^BCR-ABL^-transformed cells with WT CDK6, CDK6K43M, or CDK6R31C. Several independently derived cell lines were injected subcutaneously into mice and the ability to stimulate angiogenesis was found to correlate with CDK6 expression, independently of whether the CDK6 expressed was WT or a mutant version (Figures 5A and 5B). Tumor growth inversely correlated with the ability of the CDK6 construct to induce and/or bind to p16^INK4a^ (Figure 5C). A similar correlation between CDK6 and angiogenesis could be verified in *NPM-ALK*^*+*^ tumors when we subcutaneously injected *NPM-ALK*^*+*^ *Cdk6*^−*/*−^ and *NPM-ALK*^*+*^ *Cdk6*^*+/+*^ cell lines into recipient mice. *Cdk6*^−*/*−^ tumors evolved significantly later and displayed severely reduced blood vessel formation (Figures S5E and S5F). To further confirm the pro-angiogenic effect of CDK6, we added the cell supernatant of the *Cdk6*-deficient p185^BCR-ABL^-transformed cells expressing WT CDK6, CDK6K43M or CDK6R31C to murine endothelial cells. Supernatant from cells expressing CDK6—either WT or mutant—was capable of significantly stimulating endothelial cell proliferation, migration, and sprouting (Figures 5D–5G). Collectively, these data link high CDK6 expression to enforced tumor angiogenesis.

### Transcriptional Regulation of *Vegf-A* by CDK6

**Figure 6:**
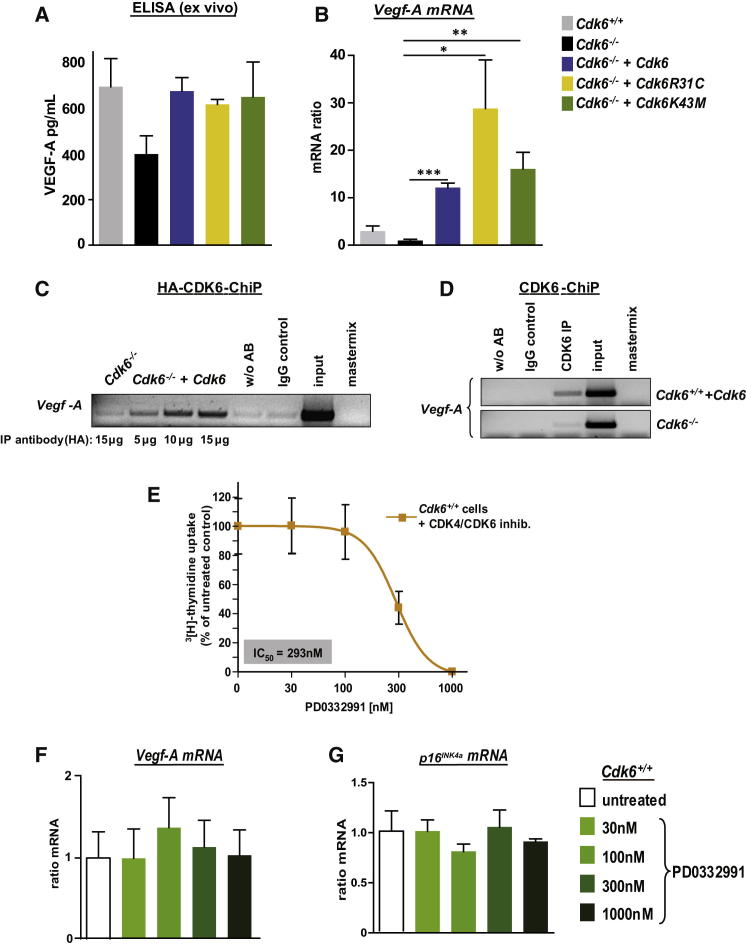
CDK6 Regulates Transcription of the Pro-Angiogenic Factor *Vegf-A* (A) Ex vivo VEGF-A protein levels (pg/ml) of the subcutaneous tumors (see Figures 5A–5C) were analyzed with an ELISA experiment (n = 3). (B) Relative *Vegf-A* mRNA levels of indicated cells were analyzed by qPCR. The fold change compared to *Cdk6*^−*/*−^*Vegf-A* mRNA level is shown (n ≥ 4; *Cdk6*^−*/*−^ versus: *Cdk6*^−*/*−^*+Cdk6*, ^∗∗∗^p < 0.0001; *Cdk6*^−*/*−^*+Cdk6R31C*, ^∗^p = 0.03; *Cdk6*^−*/*−^*+Cdk6K43M*, ^∗∗^p = 0.004). (C and D) ChIP assays were performed on (C) *Cdk6*^−*/*−^ and *Cdk6*^−*/*−^ cells expressing an HA-tagged CDK6 using different amounts of an anti-HA-antibody as well as on (D) *Cdk6*^−*/*−^ and *Cdk6*^*+/+*^*+Cdk6* cells using an anti-CDK6-antibody. PCR was performed to detect the *Vegf-A* promoter sequence. (E) Dose-response curve of *Cdk6*^*+/+*^ p185^BCR-ABL^-transformed cells treated 24 hr with the CDK6/4 inhibitor PD0332991 (n = 3). (F) *Vegf-A* mRNA levels of *Cdk6*^*+/+*^ p185^BCR-ABL^-transformed cells (n = 3) treated 24 hr with 0, 30, 100, 300, and 1000 nM PD0332991 were analyzed by qPCR. The fold change compared to untreated *Vegf-A* mRNA levels is shown. (G) *p16*^*INK4a*^ mRNA levels of *Cdk6*^*+/+*^ p185^BCR-ABL^–transformed cells (n = 3) treated 24 hr with 0, 30, 100, 300, and 1,000 nM PD0332991 were analyzed with qPCR. The fold change compared to untreated *p16*^*INK4a*^ mRNA levels is shown. Error bars indicate the mean ± SEM. See also Figure S6.

Because tumor angiogenesis is frequently driven by ligands of the vascular endothelial growth factor (VEGF) receptor, we investigated the levels of the most prominent growth factor VEGF-A (Ferrara and Davis-Smyth, 1997, Neufeld et al., 1999). Similiar to our observations on the CDK6-mediated control of p16^INK4a^, we found that CDK6 induced the expression of *Vegf-A* pre-mRNA, mRNA, and protein, irrespective of the mutant used (Figures 6A and 6B; Figures S6A–S6E). Enforced expression of CDK4 did not induce *Vegf-A* and no changes in angiogenesis could be detected in CDK4-high tumors (Figures S6F–S6H), confirming the functional differences between CDK6 and CDK4. ChIP assays verified CDK6’s role in the regulation of *Vegf-A* transcription in *Cdk6*^−*/*−^ p185^BCR-ABL^-transformed cells expressing an HA-tagged CDK6 as well as in *Cdk6*^*+/+*^*+Cdk6* cells (Figures 6C and 6D; Figure S6I). Increased CDK6-induced *Vegf-A* transcription took place via a significant increase in H3K36 trimethylation; changes to other histone marks were not statistically significant (Figures S6J and S6K).

A small molecule inhibitor directed against the ATP binding pocket domain of CDK4 and CDK6 is currently being tested in clinical trials (Fry et al., 2004). When we treated cell lines expressing WT CDK6 with the CDK6/CDK4 inhibitor (PD0332991), we found a significant reduction of cell proliferation. In contrast, inhibitor treatment failed to affect the levels of *Vegf-A* or *p16*^*INK4a*^ mRNA, confirming that CDK6’s role in transcriptional control is independent of its kinase activity (Figures 6E–6G).

The regulation of VEGF-A by CDK6 was not restricted to *BCR-ABL*^*+*^ cells, but was also observed in our T cell lymphoma model. *NPM-ALK*^*+*^ *Cdk6*^−*/*−^ cells showed drastically reduced *Vegf-A* mRNA levels compared to *NPM-ALK*^*+*^ *Cdk6*^*+/+*^ cells (Figure S6E). Similarly, the investigation of 23 human B- and T-lymphoid cell lines revealed the positive correlation between *VEGF-A* and *CDK6*, but not *CDK4* mRNA expression levels (Figures S6L–S6O).

### Interaction Partners of CDK6

**Figure 7:**
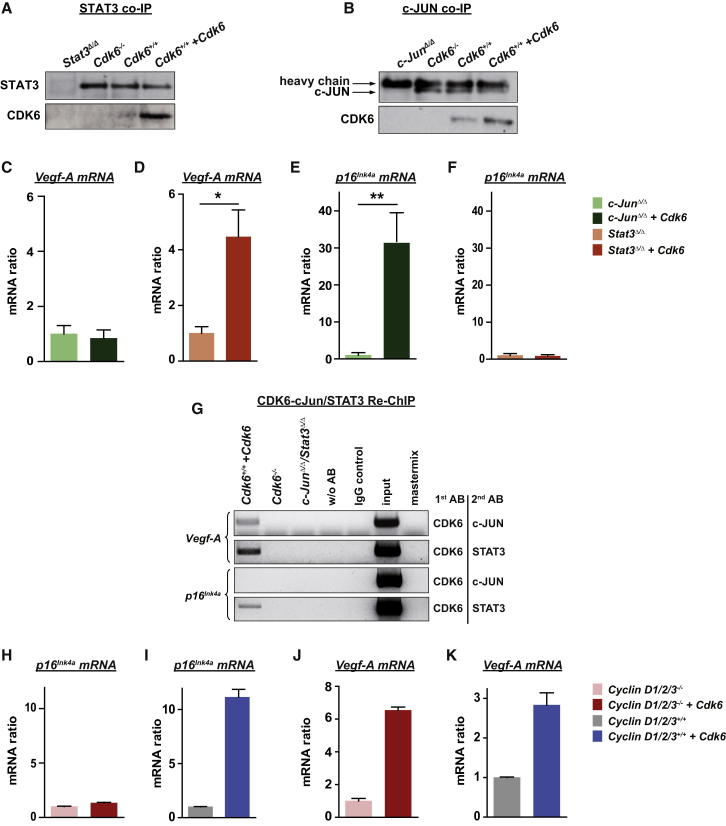
Transcriptional Interaction Partners of CDK6 (A) An anti-STAT3 co-immunoprecipitation (co-IP) was performed with *Stat3*^Δ/Δ^, *Cdk6*^−*/*−^, *Cdk6*^*+/+*^, and *Cdk6*^*+/+*^*+Cdk6* cell extracts and immunoblotted for STAT3 and CDK6. (B) An anti-c-JUN co-IP was performed with *c-Jun*^Δ/Δ^, *Cdk6*^−*/*−^, *Cdk6*^*+/+*^, and *Cdk6*^*+/+*^*+Cdk6* cell extracts and immunoblotted for c-JUN and CDK6. (C and D) *Vegf-A* mRNA levels of (C) *c-Jun*^Δ/Δ^ versus *c-Jun*^Δ/Δ^*+Cdk6* and (D) *Stat3*^Δ/Δ^ versus *Stat3*^Δ/Δ^*+Cdk6* p185^BCR-ABL^-transformed cells were analyzed with qPCR (n ≥ 3; *Stat3*^Δ/Δ^*versus Stat3*^Δ/Δ^*+Cdk6*: ^∗^p = 0.025). (E and F) *p16*^*INK4a*^ mRNA levels of (E) *c-Jun*^Δ/Δ^ versus *c-Jun*^Δ/Δ^*+Cdk6* and (F) *Stat3*^Δ/Δ^ versus *Stat3*^Δ/Δ^*+Cdk6* p185^BCR-ABL^-transformed cells were analyzed with qPCR (n ≥ 3; *c-Jun*^Δ/Δ^ versus *c-Jun*^Δ/Δ^*+Cdk6*: ^∗∗^p = 0.008). (G) A potential interaction between CDK6 and c-JUN or STAT3 was analyzed in *Cdk6*^*+/+*^*+Cdk6* cells with ChIP-Re-ChIP experiments at the promoter regions of *Vegf-A* and *p16*^*INK4a*^. Antibodies used for ChIP (1st AB) and Re-ChIP (2nd AB) are shown on the right. (H–K) *Vegf-A* (H and I) and *p16*^*INK4a*^ (J and K) mRNA levels of Cyclin D1/2/3^−*/*−^ MEFs versus cyclin D1/2/3^−*/*−^ MEFs enforced expressing CDK6 as well as cyclin D1/2/3^+/+^ MEFs versus cyclin D1/2/3^+/+^ MEFs enforced expressing CDK6 were analyzed with qPCR. Error bars indicate the mean ± SEM. See also Figure S7.

**Figure 8:**
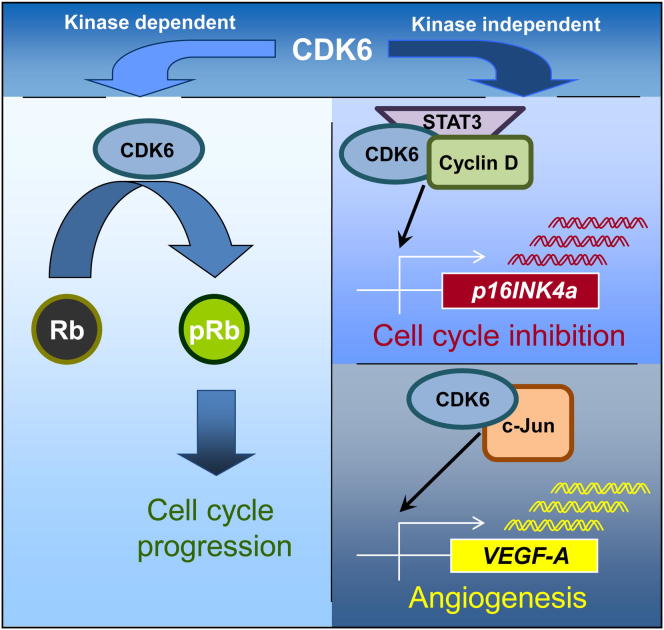
Schematic Representation of the Kinase-Dependent and the Noncanonical Kinase-Independent Functions of CDK6

STAT and AP-1 transcription factors are key components for the progression of *BCR-ABL*^*+*^ leukemia as well as in *NPM-ALK*^*+*^ lymphomagenesis. Both pathways are known to regulate expression of p16^INK4a^ as well as of VEGF-A (Kollmann et al., 2011b, Niu et al., 2002, Passegué and Wagner, 2000, Schmidt et al., 2007, Yin et al., 2009). Co-immunoprecipitation experiments revealed that CDK6, but not CDK4, is in a complex with the proto-oncogene STAT3 and the AP-1 transcription factor c-JUN (Figures 7A and 7B; Figure S7A). To test whether the transcriptional activity of CDK6 depends on these proteins, we used p185^BCR-ABL^-transformed cell lines lacking either c-JUN or STAT3. CDK6 was capable of inducing VEGF-A expression in the absence of STAT3, but not in the absence of c-JUN (Figures 7C and 7D). In contrast, induction of *p16*^*INK4a*^ by CDK6 required the presence of STAT3 (Figures 7E and 7F). The findings were confirmed with ChIP/Re-ChIP studies (Figures 7G, S7B, and S7C). Recent studies have shown that D-type cyclins have transcriptional activity (Bienvenu et al., 2010, Despouy et al., 2003, Musgrove et al., 2011). In our experimental system, cyclin D2 and cyclin D3 are the interaction partners of CDK4 and CDK6, while cyclin D1 is not expressed (Figure S7D and data not shown). ChIP/Re-ChIP experiments confirmed that cyclin D2 and CDK6 are present as a complex at the *p16*^*INK4a*^ and the *Vegf-A* promoters, and CDK6 is also present at the *p16*^*INK4a*^ promoter without D-type cyclins (Figures S7E and S7F). Interestingly, CDK6 failed to induce *p16*^*INK4a*^ transcription in MEFs deficient for *cyclins D1, D2*, and *D3* (Figures 7H and 7I), but retained the ability to induce *Vegf-A* transcription (Figures 7J and 7K). We failed to detect cyclin D3 at the *p16*^*INK4a*^ and the *Vegf-A* promoters, but can currently not exclude that this is due to the lack of an antibody suitable for ChIP experiments. In summary, our data show that CDK6 is contained in transcriptional complexes that include STAT transcription factors and D-type cyclins. Transcription of different target genes depends on different members of the complexes. CDK6 may regulate transcription in at least two distinct ways—either in cooperation with STAT3 and D-type cyclins, e.g., to induce *p16*^*INK4a*^ expression, or together with the AP-1 transcription factor c-JUN, when it upregulates VEGF-A (Figure 8).

## Discussion

CDK6, but not its close homolog CDK4, is frequently expressed at high levels in human and murine lymphoma and leukemia and has been proposed to be a driving force for these diseases. Despite the overwhelming evidence that CDK6 has a unique role, the underlying mechanism for the preferential upregulation of CDK6 has remained enigmatic. In this study, we show that CDK6, in addition to its role as a cell-cycle kinase, has important kinase-independent functions as a transcriptional regulator. The nonredundant role of CDK6 in promoting tumors in B and T cell malignancies is underlined by the strong delay in latency observed in *Cdk6*-deficient animals in both NPM-ALK- and BCR-ABL-driven diseases.

The enforced expression of CDK6 in in vitro-generated p185^BCR-ABL^-transformed cell lines unmasked an inhibitory effect on cell proliferation. On first thought, the result might appear paradoxical, but our findings can be rationalized by postulating a feedback loop including the upregulation of the tumor suppressor and cell-cycle inhibitor p16^INK4a^. Although p16^INK4a^ is also able to promote oncogene-induced senescence (Campisi, 2012), we failed to obtain any evidence of senescence. CDK4 and CDK6 have recently been shown to counteract the induction of senescence by phosphorylating FOXM1, which may contribute to the lack of senescence induction (Anders et al., 2011). Confirmation that p16^INK4a^ is involved in CDK6’s tumor-suppressing role was obtained from experiments with *p16*^*INK4a*^*/p19*^*ARF*^-deficient animals and the CDK6R31C mutant unable to bind INK proteins. CDK6 combines two opposing functions: it is able to inhibit or accelerate cell proliferation depending on whether p16^INK4a^ is present or not. Levels of CDK6 under physiologic conditions are confined to a narrow window determined by these two opposing effects on cell growth.

Accordingly, the requirement for p16^INK4a^ may be regarded as an internal safeguard that needs to be overcome to allow CDK6 to exert its tumor-promoting function. Malignancies of the B- or T-lymphoid lineage frequently display loss of *p16*^*INK4a*^ due to deletion, mutation, or methylation (Fizzotti et al., 1995, Hackanson et al., 2005, Hebert et al., 1994, Hennessy et al., 2003, Kees et al., 1997, Malumbres and Barbacid, 2001, Nobori et al., 1994, Ruas and Peters, 1998). We propose that CDK6 only confers a proliferative advantage to a transformed cell in the absence of p16^INK4a^. Support for this hypothesis is provided by our analysis of histologic sections of human lymphoid malignancies, which show an inverse correlation of CDK6 and p16^INK4a^ expression. Histologic analysis allowed the determination of p16^INK4a^ and CDK6 expression at the level of individual cells. This is crucial when studying diseases such as NPM-ALK-driven lymphoma, in which malignant cells are embedded within a microenvironment containing normal nontransformed lymphoid cells.

Our data predict that enhanced levels of CDK6 protein drive both cell-cycle progression and angiogenesis. It is currently unclear what causes the initial upregulation of CDK6 and the silencing of *p16*^*INK4a*^. It is likely that several events are required, possibly including aberrant expression of microRNAs, which have been reported to regulate CDK6 expression. Subsequent deletion or methylation of *p16*^*INK4a*^ may confer a clonal advantage to the cell and induce a selection process that allows the outgrowth of a distinct CDK6 high p16^INK4a^-deleted clone. The ability to promote angiogenesis provides a rationale for the preferential growth of cells that express high levels of CDK6. CDK6 is not the only factor that promotes blood vessel growth, but it contributes substantially to meeting the tumor’s enhanced demand for blood supply.

Two important functions have been assigned to cyclin D/CDK4 (DK4) and cyclin D/CDK6 (DK6) complexes, i.e., phosphorylation of the retinoblastoma (Rb) protein and sequestering of p21^Cip^ and p27^Kip1^. Both permit progression through the G1 phase of the cell cycle. DK4 and DK6 have been considered functionally redundant in advancing the cell cycle. However, CDK6 has recently been proposed to have additional tasks; for example, it has been implicated in the differentiation of cell types such as astrocytes and T cells (Ericson et al., 2003, Grossel and Hinds, 2006, Hu et al., 2009). We speculate that CDK6’s function in linking growth and differentiation is also (at least partially) mediated by kinase-independent functions. Multicellular organisms could not have evolved without a linkage between growth and differentiation, so CDK6 may be part of a control mechanism to ensure the generation of complex and fully functional organisms. It is of particular interest that recent findings implicate D-type cyclins in the link between cell-cycle control, differentiation, and DNA damage repair (Jirawatnotai et al., 2011, Li et al., 2010). CDK6 and D-type cyclins may even act in concert to link cell-cycle control to other cellular functions, permitting both versatility and the possibility of fine-tuning by the inclusion of components specific for particular stages in the cell cycle.

We are only now starting to understand the importance of the different roles of cell-cycle regulators, so it is likely that CDK6 has additional functions in body homeostasis. Uncovering such roles is hampered by the fact that *Cdk6* knockout mice are viable, although this does not exclude the possibility that the CDK6 protein may be important under particular circumstances or that the absence of CDK6 may be compensated for by other factors during development. Tumorigenesis is one such condition in which cell signaling is rewired and previously redundant signaling pathways assume much greater significance and turn into key signaling nodes. There is a single report that the CDK6 protein is part of a complex that binds DNA in an androgen receptor-dependent manner to enhance transcription of the prostate-specific antigen in LNCaP prostate cancer cells (Lim et al., 2005). Our results show that CDK6 is also a component of a transcription complex that includes a variety of transcription factors. CDK6 may act in concert with STAT3 to induce p16^INK4a^ expression, or with AP-1 transcription factors to upregulate VEGF-A. Interestingly, VEGF-A regulation seems to be independent of D-type cyclin binding whereas in the absence of cyclins, CDK6 is unable to induce p16^INK4a^ expression. Although transcription of different genes requires different factors, our ChIP experiments strongly suggest that all components of the CDK6-STAT-cyclin D complex are present at all promoters, even if they are not essential for transcription. Further work will be required to identify CDK6’s interaction partners in different cell types and to understand the molecular mechanisms that determine the importance of the binding of cyclins and other interaction partners for distinct transcriptional targets.

In the absence of p16^INK4a^, elevated CDK6 levels promote tumorigenesis. For example, in lymphoma CDK6 induces the transcription of growth factors such as *Vegf-A.* Although tumor growth depends on angiogenesis, there are few indications of factors involved in the regulation of both processes. The mechanism underlying their interdependence has thus remained elusive. Because CDK6 promotes tumor growth while simultaneously guaranteeing the supply of oxygen and energy to the rapidly growing tumor, it enables tumor cells to proliferate extremely efficiently.

Considerable effort has been expended on targeting angiogenesis for the treatment of tumors. These methods suffer from the potential limitation that they may promote the appearance of highly malignant metastatic subclones that evolve under hypoxic pressure resulting from treatment with angiogenic inhibitors. A targeted therapy against CDK6, simultaneously inhibiting growth and angiogenesis, may represent a unique opportunity to overcome the detrimental effects. CDK6 kinase activity already represents a promising target for anticancer drugs (Choi et al., 2012, Sawai et al., 2012). In light of our findings, it is important that future drug design take into account the kinase-independent function of the CDK6 protein. Because CDK6 knockout mice are viable, such inhibitors should have high antitumor effects and be specific for tumorigenesis. Our findings also suggest that CDK6-directed therapies may be useful for *Rb* deficient tumors that do not rely on the cell cycle effects of CDK4/CDK6 complexes.

## Experimental Procedures

### Mice

All mice were on a C57BL/6 background. *Cdk6*^−*/*−^ (Malumbres et al., 2004), *INK4a/ARF*^−*/*−^ (Serrano et al., 1996), *NPM-ALK-*tg (Chiarle et al., 2003), *Nu/Nu*, and *Rag2*^−*/*−^ mice (Shinkai et al., 1992) have been described previously. Animal experiments were performed in accordance with protocols approved by the Animal Welfare Committee at the Medical University of Vienna.

### Handling of Human Subjects

The B-lymphoid tissue array was obtained from the Institute of Pathology, University Hospital Graz. The T-lymphoid tissue array was obtained from the Department of Clinical Pathology, University of Vienna and was established using samples from patients with anaplastic large cell lymphoma (NPM-ALK positive and NPM-ALK negative). Human peripheral blood samples were recruited for this study from the NHS blood and transplant (NHSBT) in Cambridge, UK. Samples were obtained after informed consent in compliance with the Declaration of Helsinki. All samples were de-identified prior to analysis.

### Monolayer Wounding Assay

In this assay, 1 × 10^5^ murine endothelial cells (mECs) were seeded in endothelial cell growth medium (RPMI containing 10% fetal calf serum (FCS) mixed 1:1 with Epithelial Cell Growth Medium MV (PromoCell). After 24 hr, medium was removed and a scratch made through the monolayer. Cells were washed twice with PBS and covered with supernatants as appropriate.

For supernatant production, a defined cell number of different cell lines was incubated in medium for 24 hr. Supernatant was collected and filtered to remove leukemic cells.

After 2- and 24-hr incubation of mECs with the supernatants, pictures of the scratch were taken under a microscope (Nikon, Eclipse TS100; 10×) using a digital camera (Nikon, Coolpix P5000; Zoom F2.7). To analyze migration differences, the percentage of the open area of the scratch was measured with TScratch software.

### Spheroid Sprouting Assay

mECs were suspended in 80% endothelial cell growth medium (RPMI containing 10% FCS mixed 1:1 with Epithelial Cell Growth Medium MV (PromoCell) and 20% methylcellulose (Sigma) and seeded as drops (800 cells/30 μl) in nonadherent dishes. The dishes were incubated upside down as hanging drops for 24 hr. Under these conditions, all suspended cells contribute to the formation of a single spheroid per drop of defined size and cell number (800 cells/spheroid). Spheroids were harvested and seeded in methylcellulose and supernatant (prepared as described under Monolayer Wounding Assay) of the leukemic cells was added. After 24-hr incubation, the cumulative length of the sprouts from each spheroid was measured with ImageJ software.

### ChIP and Re-ChIP Assays

ChIP assays were performed using the chromatin immunoprecipitation assay kit (Upstate Biotechnology) according to the manufacturer’s protocol: 5 × 10^6^ cells were treated with 1% formaldehyde for 10 min and then lysed. Chromatin was sheared to 200–1,000 bp fragments using Bioruptor (Diagenode). The antibodies are given in the Supplemental Experimental Procedures. Nonrelated IgG (10 μg) was used as a control. Immunoprecipitated DNA was phenol-chlorophorm-extracted, ethanol-precipitated, and dissolved in 30 μl Tris-EDTA (TE) buffer. Two microliters of recovered DNA were used for subsequent PCR analysis. Primer sequences for *p16*^*INK4a*^, *ARF*, and *Vegf-A* promoter and PCR conditions were as reported previously (Gonzalez et al., 2006, Ray et al., 2007). PCR products were separated on a 2% agarose gel stained with GelRed (Biotium).

For Re-ChIP experiments, 1 × 10^8^ cells were lysed and chromatin-sonicated as described above. After immunoprecipitation with the first antibody, protein-DNA complexes were eluted from protein A-agarose beads by incubation for 30 min at 37°C in 50 μl of elution buffer (1× TE, 15% dithiothreitol, and protease inhibitors). The second immunoprecipitation was performed as described above.

### Statistical Analysis

Data are reported as mean values ± SEM. Biochemical experiments were performed in triplicate and a minimum of three independent experiments were evaluated. Differences were assessed for statistical significance by an unpaired two-tailed t test, by the log rank test (for Kaplan-Meier plots), or by the χ^2^-square test (for contingency tables). The p values are considered as follows: ^∗^p < 0.05; ^∗∗^p < 0.01; and ^∗∗∗^p < 0.001.

## Supplemental Information

Document S1. Supplemental Experimental Procedures and Figures S1–S7
