# A Kinase-Independent Function of CDK6 Links the Cell Cycle to Tumor Angiogenesis

**DOI:** 10.1016/j.ccell.2016.07.003

**Published:** 2016-08-08

**Authors:** Karoline Kollmann, Gerwin Heller, Christine Schneckenleithner, Wolfgang Warsch, Ruth Scheicher, Rene G. Ott, Markus Schäfer, Sabine Fajmann, Michaela Schlederer, Ana-Iris Schiefer, Ursula Reichart, Matthias Mayerhofer, Christoph Hoeller, Sabine Zöchbauer-Müller, Dontscho Kerjaschki, Christoph Bock, Lukas Kenner, Gerald Hoefler, Michael Freissmuth, Anthony R. Green, Richard Moriggl, Meinrad Busslinger, Marcos Malumbres, Veronika Sexl

(Cancer Cell *24*, 167–181; August 12, 2013)

In the original Figure 3C, the files from patient B were accidentally uploaded twice for patient B and patient C. The corrected files for patient C have been corrected in the figure below. The authors apologize for any confusion this may have caused the readers.

**Figure:**
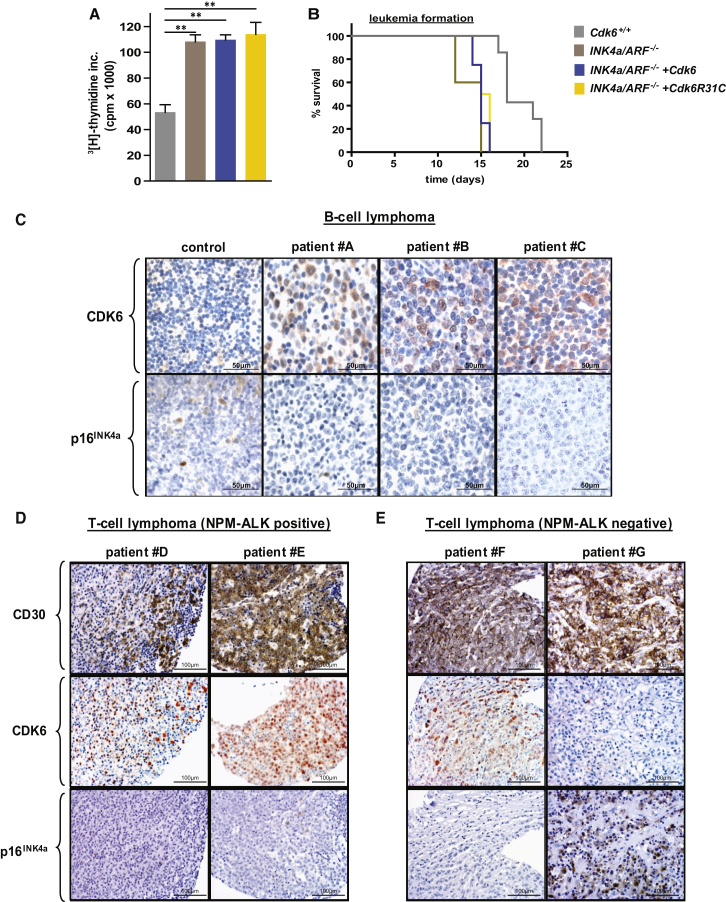
Figure 3. Inverse Relation between CDK6 and p16^INK4a^ Expression in Human Lymphomas

