# Supplementary material for: A Kinase-Independent Function of CDK6 Links the Cell Cycle to Tumor Angiogenesis
Source: Cancer Cell. 2013 Aug 12;24(2):167–81. doi: 10.1016/j.ccr.2013.07.012 (PMC3743049; doi:10.1016/j.ccr.2013.07.012)
Supplement: Document S1. Supplemental Experimental Procedures and Figures S1–S7 [file mmc1.pdf]

Cancer Cell, Volume *24*

Supplemental Information

A Kinase-Independent Function of CDK6 Links the Cell Cycle to Tumor Angiogenesis

Karoline Kollmann, Gerwin Heller, Christine Schneckenleithner, Wolfgang Warsch, Ruth Scheicher, Rene G. Ott, Markus Schäfer, Sabine Fajmann, Michaela Schlederer, Ana-Iris Schiefer, Ursula Reichart, Matthias Mayerhofer, Christoph Hoeller, Sabine Zöchbauer-Müller, Dontscho Kerjaschki, Christoph Bock, Lukas Kenner, Gerald Hoefler, Michael Freissmuth, Anthony R. Green, Richard Moriggl, Meinrad Busslinger, Marcos Malumbres, and Veronika Sexl

Inventory of Supplemental Information

- **SUPPLEMENTAL DATA**

  Figure S1, related to Figure 1.
  Figure S2, related to Figure 2.
  Figure S3, related to Figure 3.
  Figure S4, related to Figure 4.
  Figure S5, related to Figure 5.
  Figure S6, related to Figure 6.
  Figure S7, related to Figure 7.

- **SUPPLEMENTAL EXPERIMENTAL PROCEDURES**

- **SUPPLEMENTAL REFERENCES**

SUPPLEMENTAL DATA

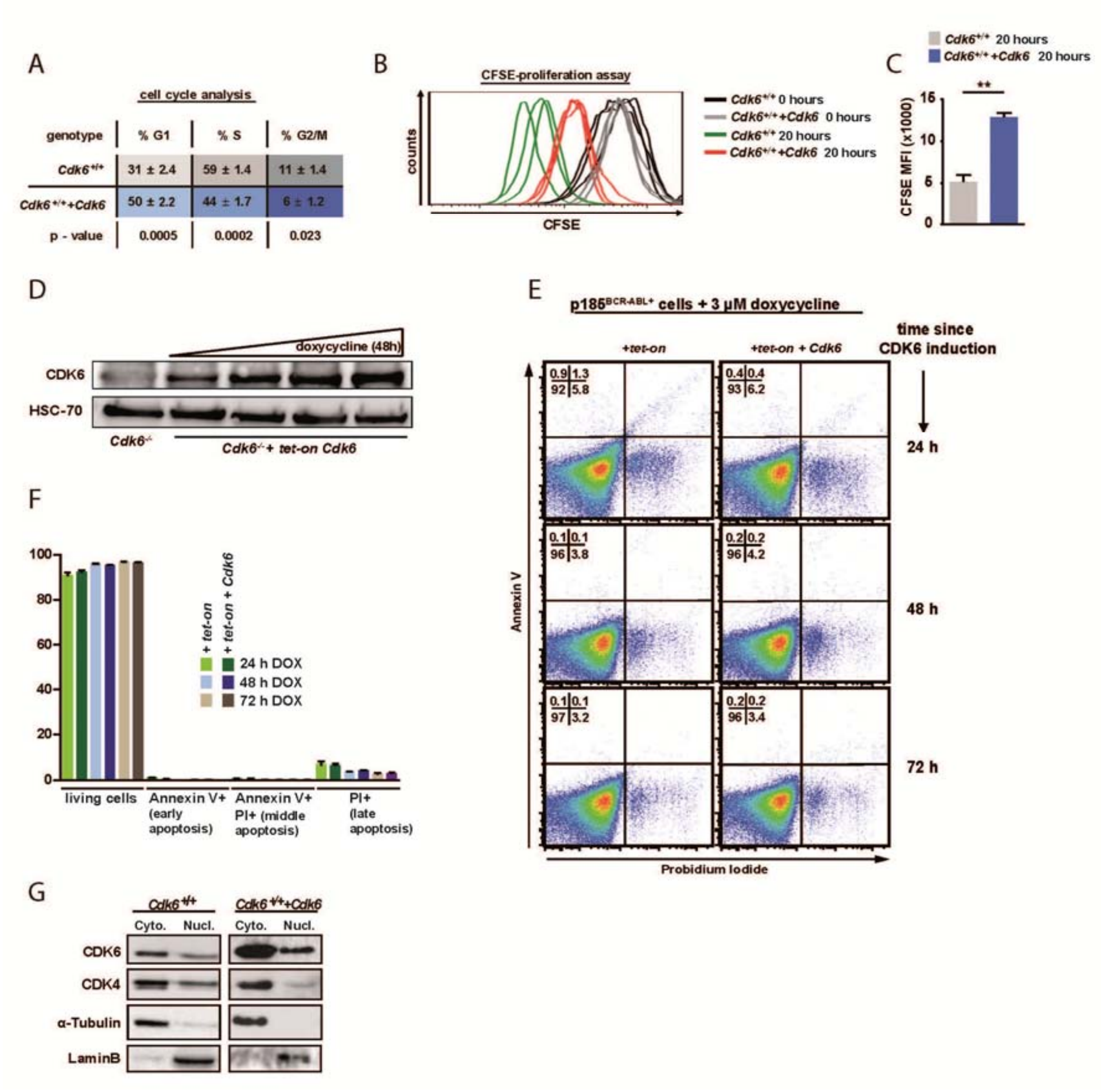

Figure S1, related to Figure 1. Enforced CDK6 expression decreases proliferation.
A) Cell cycle profiles of *Cdk6*$^{+/+}$ and *Cdk6*$^{+/+}$+*Cdk6* cells were determined by FACS (n = 3).
B) Facs-histogram overlay shows a CFSE staining to analyze proliferation of *Cdk6*$^{+/+}$ and *Cdk6*$^{+/+}$+*Cdk6* cells before and after 20 h. **C)** Proliferation assay of *Cdk6*$^{+/+}$ and *Cdk6*$^{+/+}$+*Cdk6* cells via CFSE dilution staining over a period of 20 h. Mean fluorescence intensity (MFI) was analyzed after 20 h (n = 3; p = 0.001 [**])
D) Immunoblot for CDK6 of *Cdk6*$^{-/-}$ cells expressing a doxycycline inducible *tet-on Cdk6* vector (*Cdk6*$^{-/-}$+*tet-on Cdk6*). 1.lane: *Cdk6*$^{-/-}$+*tet-on Cdk6* cells; 2.-5. lane: *Cdk6*$^{-/-}$+*tet-on Cdk6* cells 48 h after treatment with doxycycline (0.1, 0.3, 1, 3 µM).
E, F) Apoptosis stain of *Cdk6*$^{-/-}$+*tet-on Cdk6* cells 24-72h after 3µM doxycycline (DOX) treatment. Propidium iodide (PI)- annexin V+ cells, early apoptosis; PI+-annexin V+ cells,

middle apoptosis; PI+–annexinV– cells, late apoptosis. **(E)** Percentages of each apoptotic stage are in corner of each blot. **(F)** Bar graphs represent the indicated stages of apoptosis. **G)** Cytoplasmic and nuclear fractionation was performed of *Cdk6*$^{+/+}$ and *Cdk6*$^{+/+}$+*Cdk6* cells. Immunoblot for CDK6, CDK4, α-Tubulin (as cytoplasmic control) and LaminB (as nuclear control) is shown of the different fractions.

Error bars indicate the mean ± SEM.

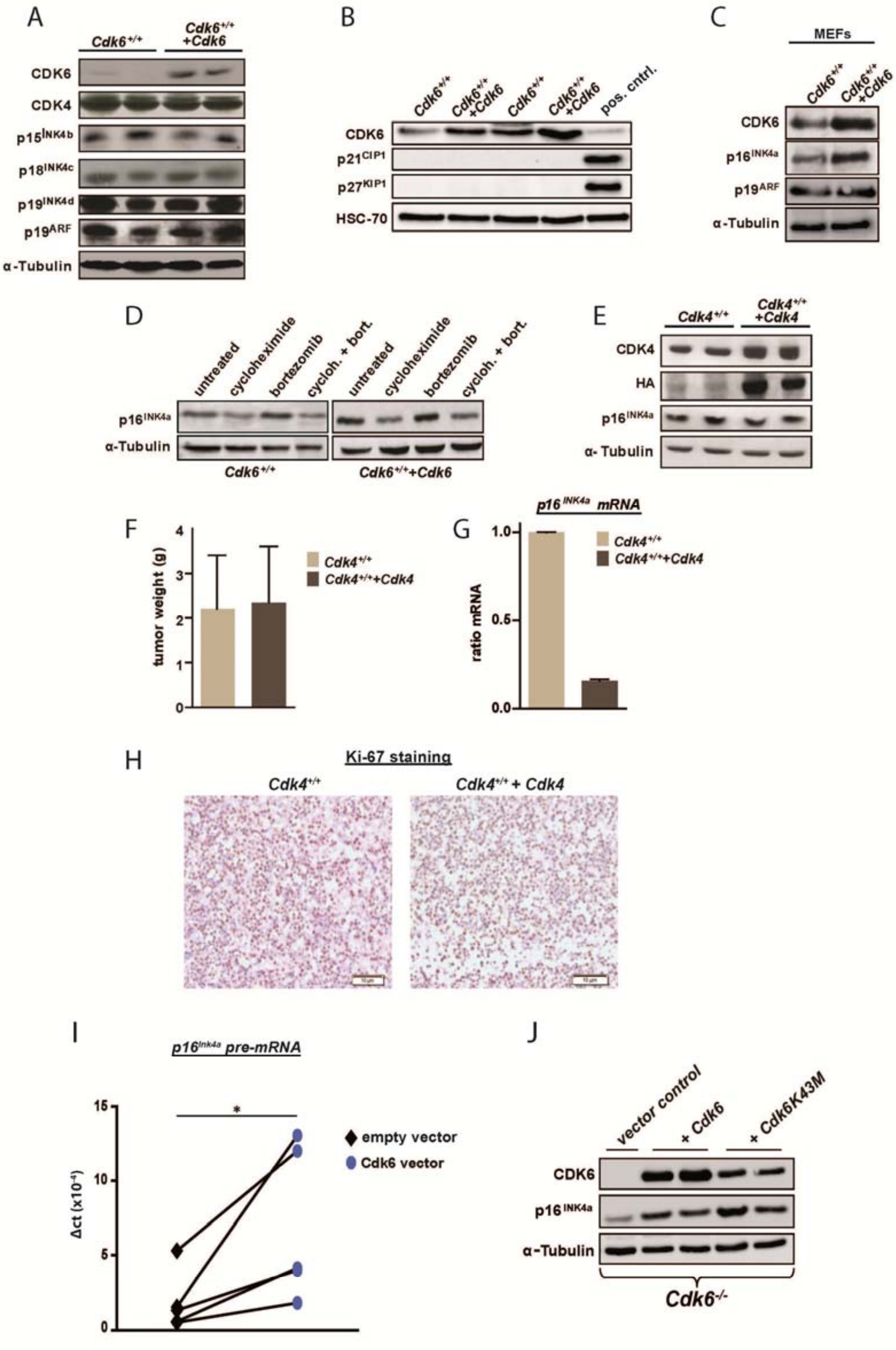
A
Cdk6+/+
Cdk6+/+ +Cdk6
CDK6
CDK4
p15INK4b
p18INK4c
p19INK4d
p19ARF
α-Tubulin
B
Cdk6+/+
Cdk6+/+ +Cdk6
Cdk6+/+
Cdk6+/+ +Cdk6
pos. cntrl.
CDK6
p21CIP1
p27KIP1
HSC-70
C
MEFs
Cdk6+/+
Cdk6+/+ +Cdk6
CDK6
p16INK4a
p19ARF
α-Tubulin
D
untreated
cycloheximide
bortezomib
cycloh. + bort.
untreated
cycloheximide
bortezomib
cycloh. + bort.
p16INK4a
α-Tubulin
Cdk6+/+
Cdk6+/+ +Cdk6
E
Cdk4+/+
Cdk4+/+ +Cdk4
CDK4
HA
p16INK4a
α- Tubulin
F
tumor weight (g)
Cdk4+/+
Cdk4+/+ +Cdk4
G
p16INK4a mRNA
ratio mRNA
Cdk4+/+
Cdk4+/+ +Cdk4
H
Ki-67 staining
Cdk4+/+
Cdk4+/+ + Cdk4
I
p16Ink4a pre-mRNA
Δct (x10-4)
empty vector
Cdk6 vector
J
vector control
+ Cdk6
+ Cdk6K43M
CDK6
p16INK4a
α-Tubulin
Cdk6-/-

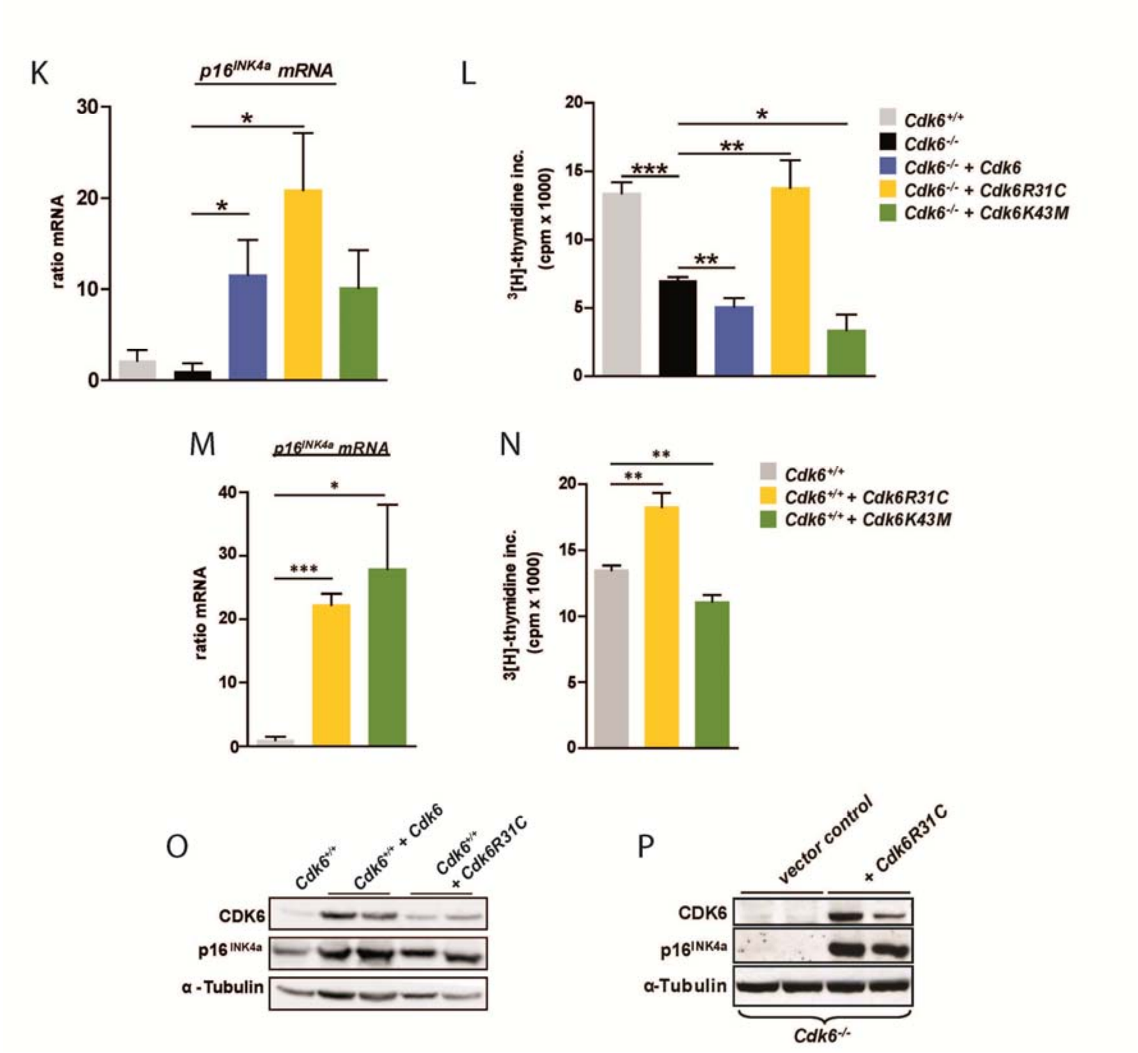

Figure S2, related to Figure 2. CDK6 regulates p16^{INK4a} expression.

A) Immunoblot for CDK6, CDK4, p15^{INK4b}, p18^{INK4c}, p19^{INK4d} and p19^{ARF} of *Cdk6*$^{+/+}$ and *Cdk6*$^{+/+}$+*Cdk6* cells.

B) Immunoblot for CDK6, p21^{CIP1}, p27^{KIP1} of *Cdk6*$^{+/+}$ and *Cdk6*$^{+/+}$+*Cdk6* cells as well as a positive control (etoposide treated p185$^{BCR-ABL}$-transformed cells as pos. cntrl. for p21^{CIP1}; murine myeloid leukemic cells as pos. cntrl. for p27^{KIP1}).

C) Immunoblot for CDK6, p16^{INK4a} and 19^{ARF} of MEFs infected with either a *pMSCV-puro* (*Cdk6*$^{+/+}$*)* or a *pMSCV-Cdk6-puro* (*Cdk6*$^{+/+}$+*Cdk6*) based retrovirus.

D) Immunoblot for p16^{INK4a} of *Cdk6*$^{+/+}$ and *Cdk6*$^{+/+}$+*Cdk6* cells after treatment with cycloheximide (an inhibitor of protein biosynthesis), bortezomib (a proteasome inhibitor) or a combination of both for four hours.

E) Immunoblot for CDK4, HA and p16^{INK4a} in p185$^{BCR-ABL}$-transformed wild type cells infected with either a *pMSCV-puro* (*Cdk4*$^{+/+}$) or a *pMSCV-Cdk4HA-puro (Cdk4*$^{+/+}$*+Cdk4)* based retrovirus.

F) *Cdk4*$^{+/+}$ and *Cdk4*$^{+/+}$+*Cdk4* cells were injected subcutaneously into *Nu/Nu* mice. Tumour weight was measured after eight days (n = 2 cell lines/genotype; n = 3 mice/genotype; p = 0.94).

G) *p16*^{INK4a} mRNA levels of *Cdk4*$^{+/+}$ and *Cdk4*$^{+/+}$+*Cdk4* cells were analysed by qPCR (n = 2).
H) Immunohistochemical stainings for the proliferation marker Ki-67 of *Cdk4*$^{+/+}$ and *Cdk4*$^{+/+}$+*Cdk4* cells. Original magnification 20x. A representative set of pictures is given.
I) *p16*^{INK4a} pre-mRNA levels of *Cdk6*$^{+/+}$ and *Cdk6*$^{+/+}$+*Cdk6 as well as Cdk6*$^{-/-}$ and *Cdk6*$^{-/-}$ +*Cdk6* were analyzed (n = 5, p = 0.048 [*]). The increased pre-mRNA levels in cells enforced expressing CDK6 do not support an altered mRNA stability in these cells.
J) Immunoblot for CDK6 and p16^{INK4a} of *Cdk6*$^{-/-}$, *Cdk6*$^{-/-}$+*Cdk6* and *Cdk6*$^{-/-}$+*Cdk6K43M* cells.
K) *p16*^{INK4a} mRNA levels of *Cdk6*$^{+/+}$, *Cdk6*$^{-/-}$, *Cdk6*$^{-/-}$+*Cdk6* and *Cdk6*$^{-/-}$+*Cdk6R31C* and *Cdk6*$^{-/-}$+*Cdk6K43M* cells were analysed by qPCR (n ≥ 4; *Cdk6*$^{-/-}$ *vs: Cdk6*$^{-/-}$+*Cdk6,* p = 0.036 [*]; *Cdk6*$^{-/-}$+*Cdk6R31C,* p = 0.02 [*]; *Cdk6*$^{-/-}$+*Cdk6K43M,* p = 0.04 [*]).
L) ^{3}[H]-thymidine incorporation of *Cdk6*$^{+/+}$, *Cdk6*$^{-/-}$, *Cdk6*$^{-/-}$+*Cdk6*, *Cdk6*$^{-/-}$+*Cdk6R31C* and *Cdk6*$^{-/-}$+*Cdk6K43M* cells was measured (n ≥ 3; *Cdk6*$^{-/-}$ vs.: *Cdk6*$^{+/+}$**:** p < 0.0001 [***]; *Cdk6*$^{-/-}$ +*Cdk6*: p = 0.008 [**]; *Cdk6*$^{-/-}$+*Cdk6R31C*: p = 0.005 [**]; *Cdk6*$^{-/-}$+*Cdk6K43M,* p = 0.04 [*]).
M) *p16*^{INK4a} mRNA levels of *Cdk6*$^{+/+}$*, Cdk6*$^{+/+}$+*Cdk6R31C* and *Cdk6*$^{+/+}$+*Cdk6K43M* cells were analysed by qPCR (n ≥ 3; *Cdk6*$^{+/+}$*vs: Cdk6*$^{+/+}$+*Cdk6R31C,* p < 0.0001 [***]; *Cdk6*$^{+/+}$+*Cdk6K43M,* p = 0.025 [*]).
N) ^{3}[H]-thymidine incorporation of *Cdk6*$^{+/+}$*, Cdk6*$^{+/+}$+*Cdk6R31C* and *Cdk6*$^{+/+}$+*Cdk6K43M* cells (n ≥ 3; *Cdk6*$^{+/+}$*vs: Cdk6*$^{+/+}$+*Cdk6R31C,* p = 0.002 [**]; *Cdk6*$^{+/+}$+*Cdk6K43M,* p = 0.006 [**]).
O) Immunoblot for CDK6 and p16^{INK4a} of *Cdk6*$^{+/+}$, *Cdk6*$^{+/+}$+*Cdk6* and *Cdk6*$^{+/+}$+*Cdk6R31C* cells.
P) Immunoblot for CDK6 and p16^{INK4a} of *Cdk6*$^{-/-}$ and *Cdk6*$^{-/-}$+*Cdk6R31C* cells.
Error bars indicate the mean ± SEM.

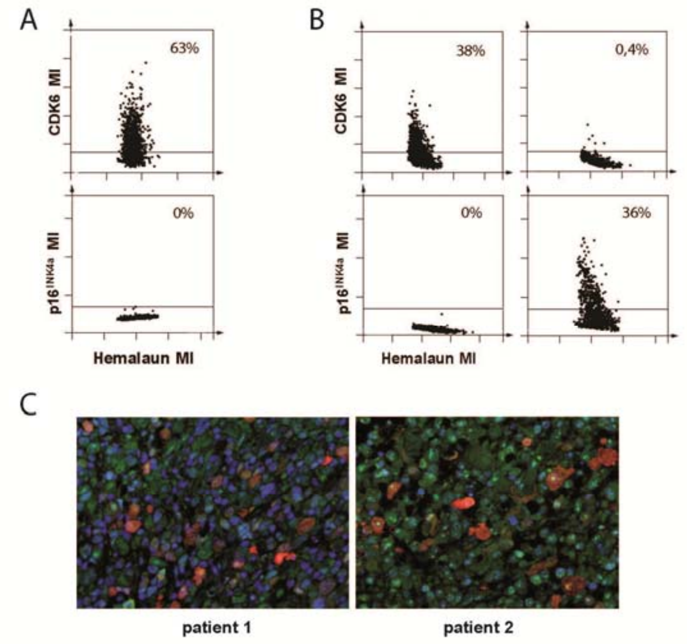

Figure S3, related to Figure 3. CDK6 and p16^{INK4a} expression in human lymphomas.

A, B) The immunohistochemical sections of **(A)** 17 *NPM-ALK* positive lymphomas and **(B)** 11 *NPM-ALK* negative lymphomas were quantified by the HistoQuest^{TM} software. Scattergrams show the percentage of cells positive for CDK6 or p16^{INK4a} of all samples (MI = Mean Intensity).

C) Double immunofluorescence stainings on 8 human ALCL whole tissue sections were performed for co-localization studies; stainings illustrated an inverse relation between CDK6 (green) and p16^{INK4a} (red), with tumour cells being either positive for CDK6 or p16^{INK4a}. Original magnification 20x. Representative cases are depicted.

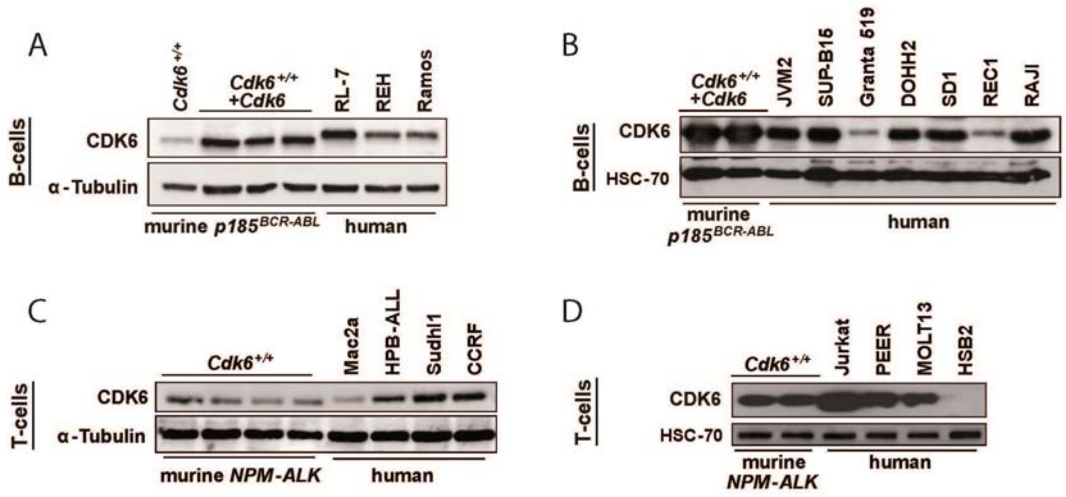

Figure S4, related to Figrues 4. CDK6 expression in murine and human cell lines.

A-D) Immunoblot for CDK6 of **(A)** murine *Cdk6*$^{+/+}$ and *Cdk6*$^{+/+}$+*Cdk6* p185$^{BCR-ABL}$-transformed cells as well as human B-lymphoid leukemic cell lines (RL-7, REH, Ramos), **(B)** murine *Cdk6*$^{+/+}$+*Cdk6* p185$^{BCR-ABL}$-transformed cells as well as human B-lymphoid leukemic cell lines (JVM2, SUP-B15, Granta 519, DOHH2, SD1, REC1, RAJI), **(C)** murine *Cdk6*$^{+/+}$ NPM-ALK-transformed cells as well as human T-lymphoid leukemic cell lines (Mac2a, HPB-ALL, Sudhl1, CCRF) and **(D)** murine *Cdk6*$^{+/+}$ NPM-ALK-transformed cells as well as human T-lymphoid leukemic cell lines (Jurkat, PEER, MOLT13, HSB2).

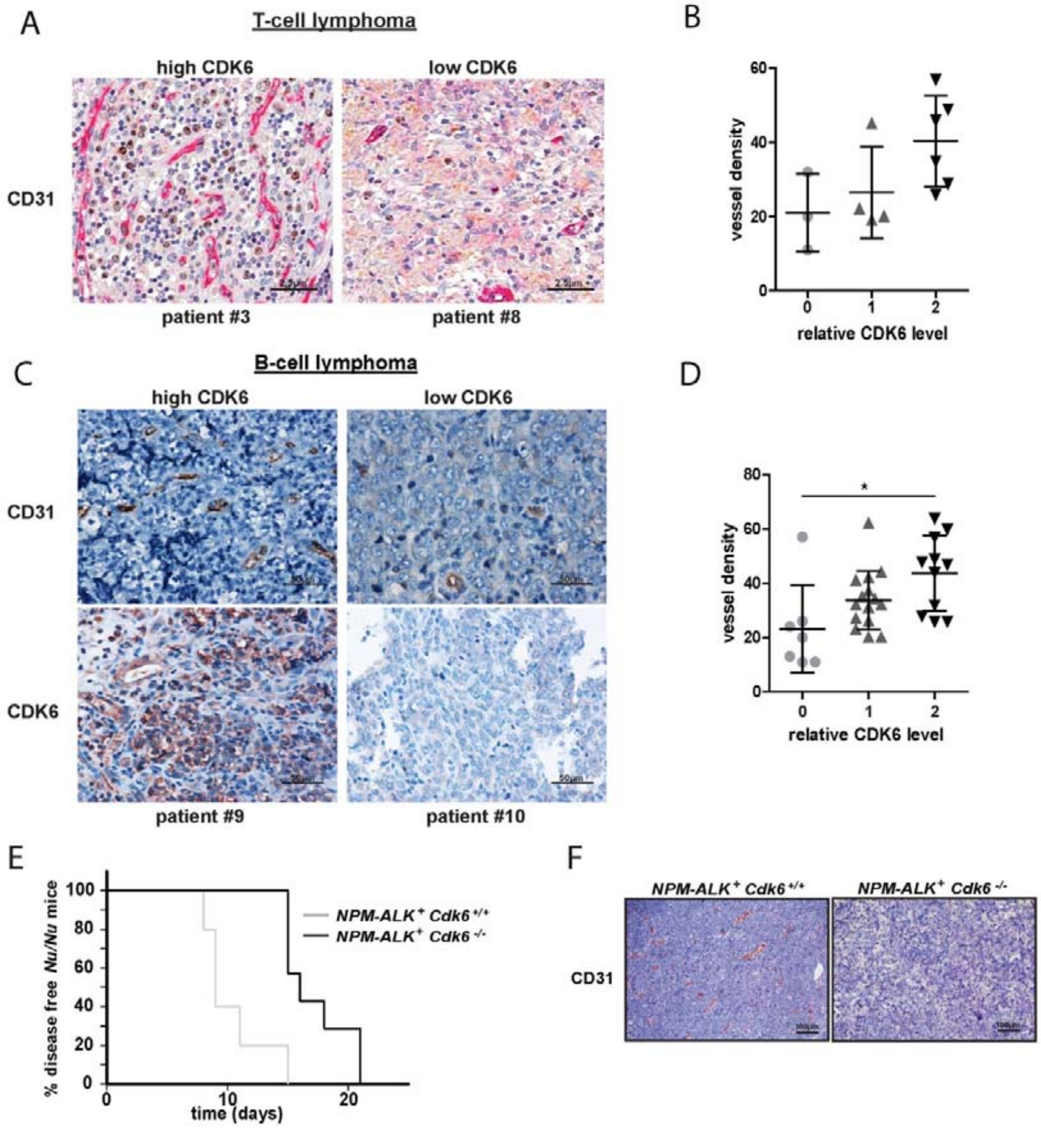

Figure S5, related to Figure 5. CDK6 regulates blood vessel formation.

A) The expression of CD31 was analysed of 13 human ALCL whole tissue sections by immunohistochemistry. Original magnification 200x. A high CDK6 expressing case (left panel, patient #3, NPM-ALK^{+}) and a low CDK6 expressing case (right panel, patient #8, NPM-ALK^{-}) are depicted.

B) Statistical analysis of **v**essel densitiy of CD31 immunohistochemical stainings of 14 human ALCL whole tissue sections (two of them depicted in panel A). Two independent observers noticed a higher vessel density in human ALCL with high CDK6 expression compared to human ALCL with low or lacking CDK6 levels. CD31 stained lumen were counted within a hotspot in an area of 0, 25 mm^2 at a magnification of 200x. Counting results confirmed the increase in vessels in T-cell lymphoma cases with high CDK6 expression.

C) The expression of CD31 and CDK6 was analysed of 33 human DLBCL whole tissue sections by immunohistochemistry. Original magnification 40x. A high CDK6 expressing

case (left panel, patient #9) and a low CDK6 expressing case (right panel, patient #10) are depicted.

D) Statistical analysis of **v**essel densitiy of CD31 immunohistochemical stainings of 33 human DLBCL whole tissue sections (two of them depicted in panel C). Two independent observers noticed a higher vessel density in human DLBCL with high CDK6 expression compared to human DLBCL with low or lacking CDK6 levels. CD31 stained lumen were counted within a hotspot in an area of 0, 25 mm^2 at a magnification of 200x. Counting results confirmed the increase in vessels in B-cell lymphoma cases with high CDK6 expression (n = 7/rel. CDK6 level 0; n = 15/rel. CDK6 level 1; n = 11/rel. CDK6 level 2; rel. CDK6 level 0 vs. rel. CDK6 level 2: p = 0.01 [*]).

E) Kaplan Meier blot of *Nu/Nu* mice subcutaneously injected with $Cdk6^{+/+}$ and $Cdk6^{-/-}$ NPM-ALK-transformed cells (n = 2 cell lines/ genotype; n = 6 mice/genotype; mean survival: 9 ($Cdk6^{+/+}$) and 16 ($Cdk6^{-/-}$); p = 0.003 [**]).

F) Immunohistochemical staining for CD31 (red) was performed in $Cdk6^{+/+}$ and $Cdk6^{-/-}$ $NPM\text{-}ALK^{+}$ subcutaneous tumours to analyze blood vessel formation. Original magnification 20x. Representative cases of each genotype are depicted.

Error bars indicate the mean ± SEM.

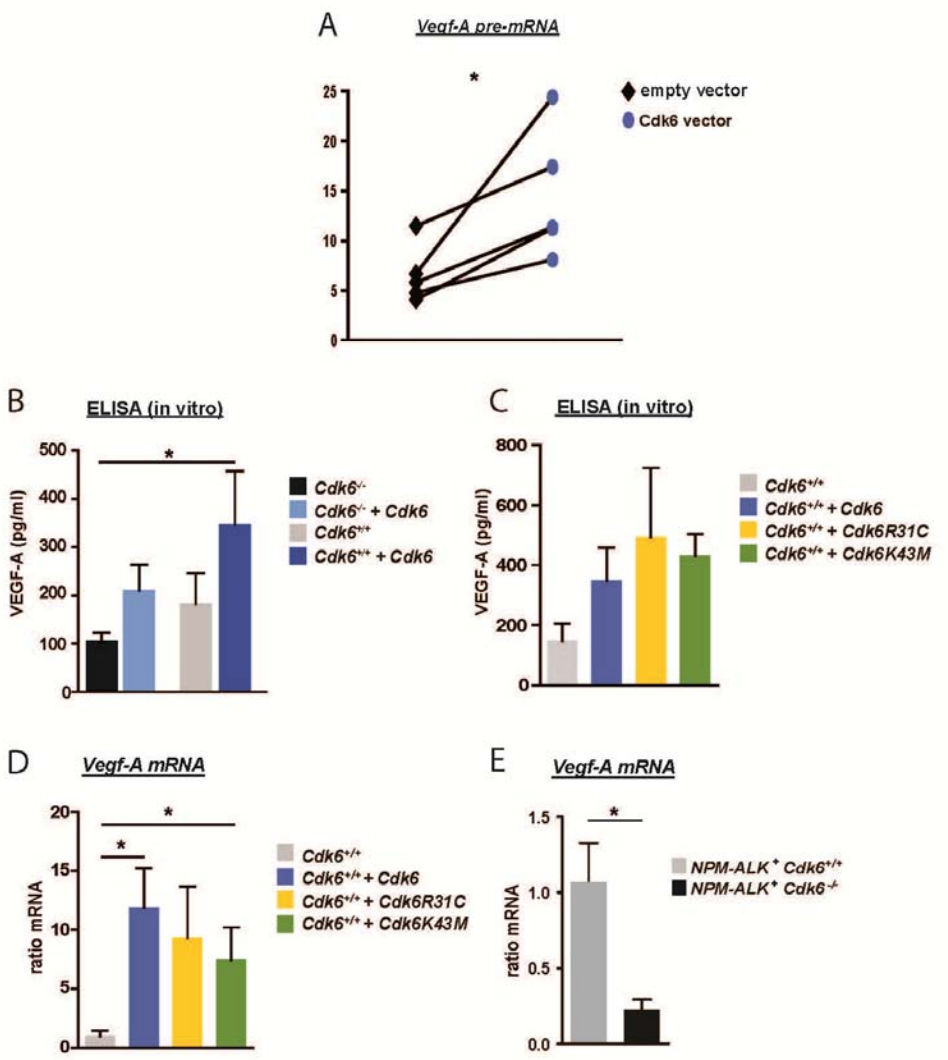
A
Vegf-A pre-mRNA
empty vector
Cdk6 vector
B
ELISA (in vitro)
VEGF-A (pg/ml)
Cdk6-/-
Cdk6-/- + Cdk6
Cdk6+/+
Cdk6+/+ + Cdk6
C
ELISA (in vitro)
VEGF-A (pg/ml)
Cdk6+/+
Cdk6+/+ + Cdk6
Cdk6+/+ + Cdk6R31C
Cdk6+/+ + Cdk6K43M
D
Vegf-A mRNA
ratio mRNA
Cdk6+/+
Cdk6+/+ + Cdk6
Cdk6+/+ + Cdk6R31C
Cdk6+/+ + Cdk6K43M
E
Vegf-A mRNA
ratio mRNA
NPM-ALK+ Cdk6+/+
NPM-ALK+ Cdk6-/-

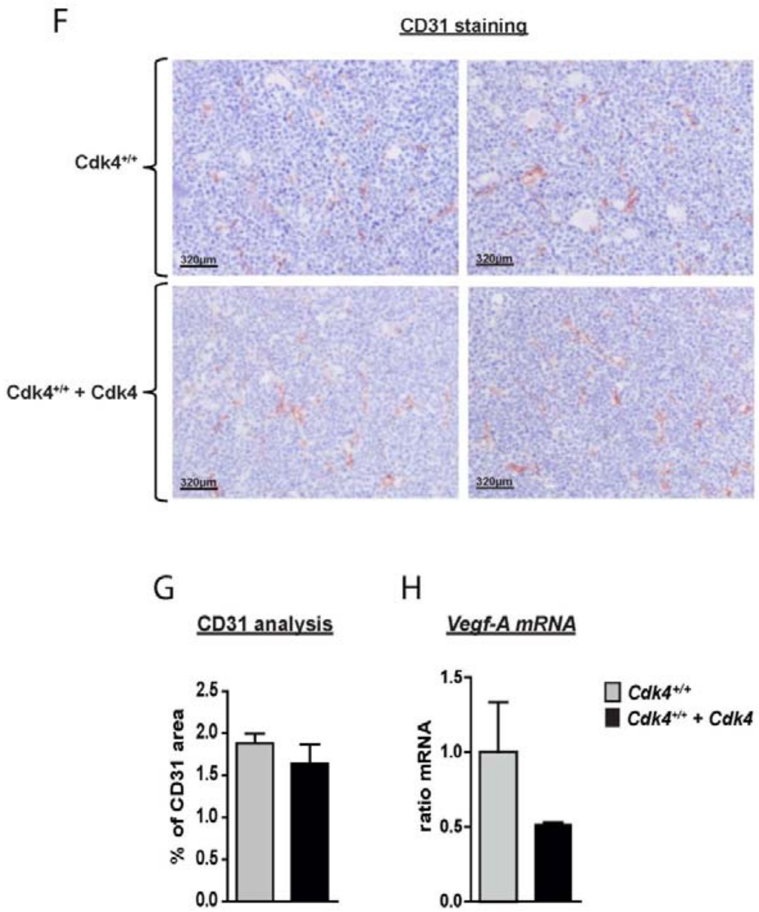
F
CD31 staining
Cdk4+/+
Cdk4+/+ + Cdk4
320µm
G
CD31 analysis
% of CD31 area
2.5
2.0
1.5
1.0
0.5
0.0
H
Vegf-A mRNA
ratio mRNA
1.5
1.0
0.5
0.0
Cdk4+/+
Cdk4+/+ + Cdk4

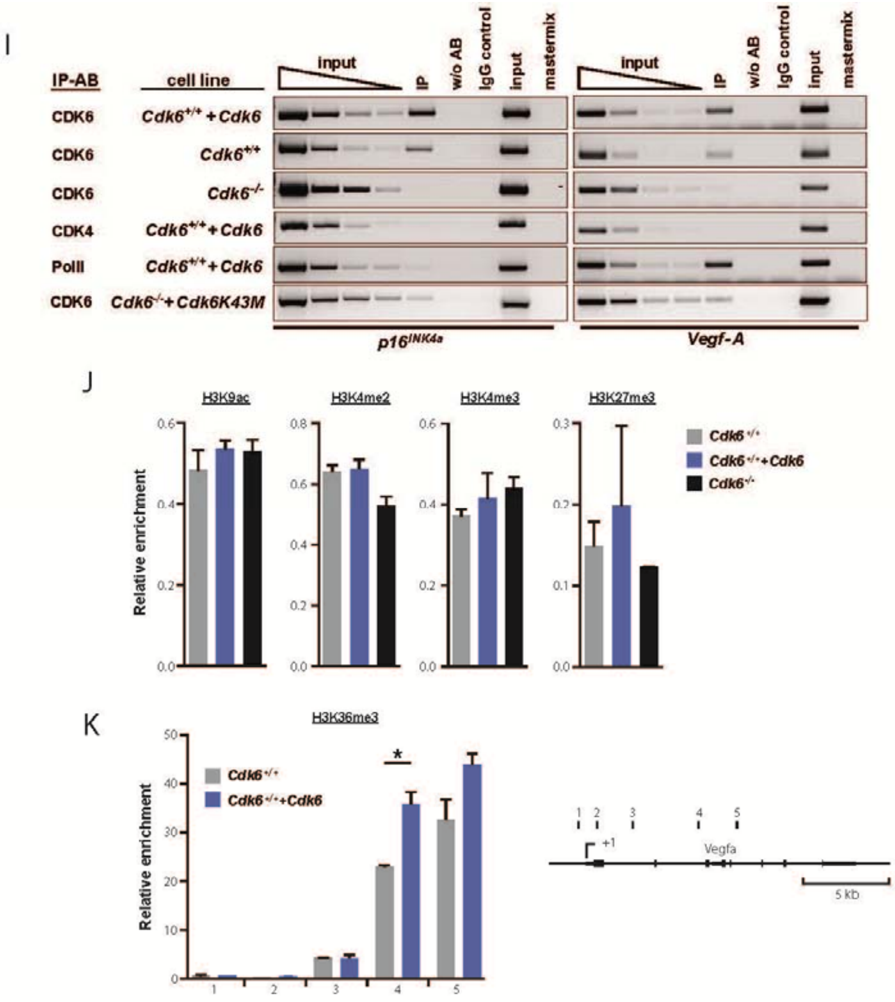
I
IP-AB
cell line
input
IP
w/o AB
IgG control
input
mastermix
CDK6
Cdk6+/+ + Cdk6
CDK6
Cdk6+/+
CDK6
Cdk6-/-
CDK4
Cdk6+/+ + Cdk6
PolII
Cdk6+/+ + Cdk6
CDK6
Cdk6-/- + Cdk6K43M
p16INK4a
Vegf-A
J
H3K9ac
H3K4me2
H3K4me3
H3K27me3
Relative enrichment
Cdk6+/+
Cdk6+/++Cdk6
Cdk6-/-
K
H3K36me3
Relative enrichment
Cdk6+/+
Cdk6+/++Cdk6
+1
Vegfa
5 kb

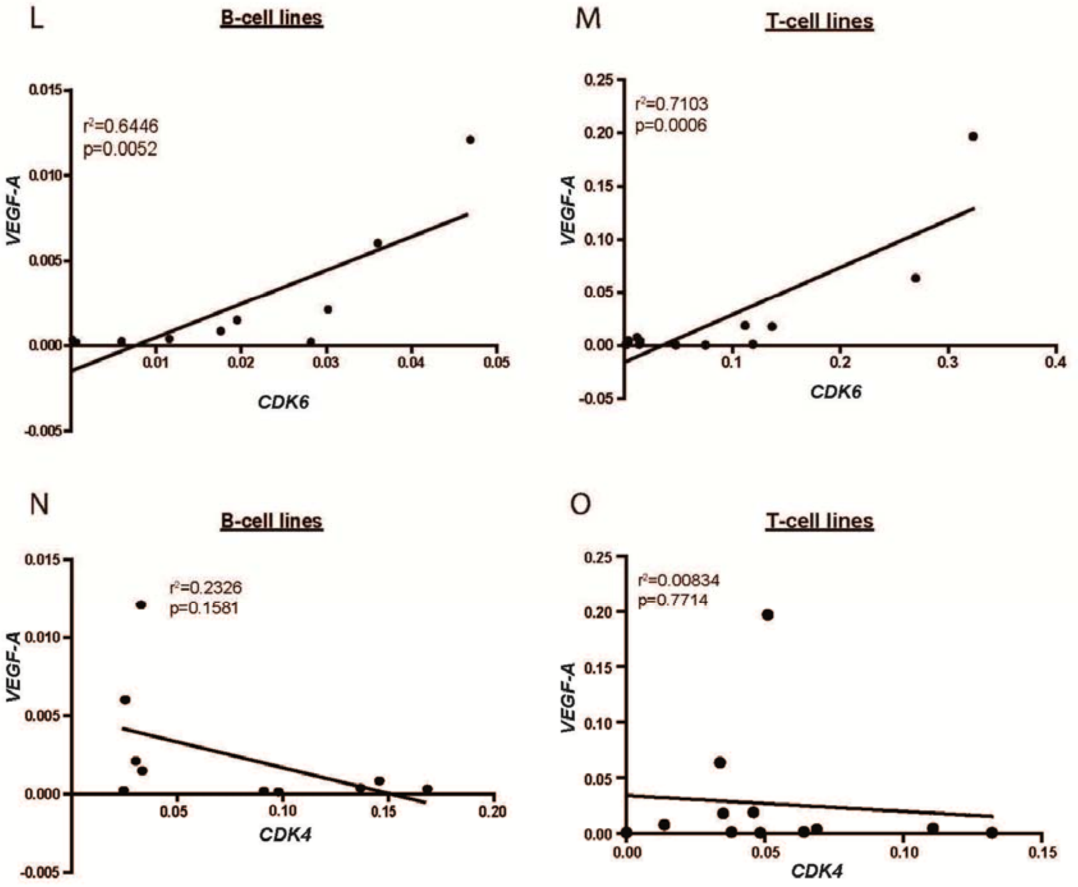

Figure S6, related to Figure 6. CDK6 regulates VEGF-A

A) *Vegf-A* pre-mRNA levels of *Cdk6*$^{+/+}$ and *Cdk6*$^{+/+}$+*Cdk6* as well as of *Cdk6*$^{-/-}$ and *Cdk6*$^{-/-}$ +*Cdk6* were analyzed (n = 5, p = 0.035 [*]). The increased pre-mRNA levels in cells enforced expressing CDK6 do not support an altered mRNA stability in these cells.

B) In vitro VEGF-A protein (pg/mL) levels in the supernatant of *Cdk6*$^{-/-}$ and *Cdk6*$^{-/-}$+*Cdk6* as well as of *Cdk6*$^{+/+}$ and *Cdk6*$^{+/+}$+*Cdk6* cells were analyzed by ELISA (n = 3; *Cdk6*$^{-/-}$ vs. *Cdk6*$^{+/+}$+*Cdk6:* p = 0.05 [*]).

C) In vitro VEGF-A protein (pg/mL) levels in the supernatant of *Cdk6*$^{+/+}$, *Cdk6*$^{+/+}$+*Cdk6, Cdk6*$^{+/+}$+*Cdk6R31C and Cdk6*$^{+/+}$+*Cdk6K43M* cells were analyzed by ELISA (n = 3).

D) *Vegf-A* mRNA levels of *Cdk6*$^{+/+}$ and *Cdk6*$^{+/+}$+*Cdk6, Cdk6*$^{+/+}$+*Cdk6R31C* and *Cdk6*$^{+/+}$+*Cdk6K43M* cells analysed by qPCR. The fold change compared to *Cdk6*$^{+/+}$ *Vegf-A* mRNA level is shown (n ≥ 3; *Cdk6*$^{+/+}$ vs.: *Cdk6*$^{+/+}$+*Cdk6*: p = 0.02 [*]; *Cdk6*$^{+/+}$+*Cdk6R31C*: p = 0.06; *Cdk6*$^{+/+}$+*Cdk6K43M*: p = 0.04 [*]).

E) *Vegf-A* mRNA levels of *Cdk6*$^{+/+}$ and *Cdk6*$^{-/-}$ NPM-ALK-transformed cells were quantified by qPCR (n = 3; p = 0.036 [*]).

F, G) *Cdk4*$^{+/+}$ and *Cdk4*$^{+/+}$+*Cdk4* p185$^{BCR-ABL}$-transformed cells were injected subcutaneously into *Nu/Nu* mice (n = 2 cell lines/genotype; n ≥ 4 tumors/genotype). **(F)** Immunofluorescence staining for CD31 (red) was performed to analyze blood vessel formation in the tumours. Original magnification 20x. Representative cases of each genotype are depicted. **(G)** Quantitative assessment (HistoQuest^{TM}) of the blood vessels of the

subcutaneous tumours (n = 3).

H) *Vegf-A* mRNA levels of *Cdk4*$^{+/+}$ and *Cdk4*$^{+/+}$+*Cdk4* cells analysed by qPCR (n = 2).

I) ChIP assays were performed using *Cdk6*$^{+/+}$+*Cdk6*, *Cdk6*$^{+/+}$, *Cdk6*$^{-/-}$ and *Cdk6*$^{-/-}$+*Cdk6K43M* cells. Protein-DNA complexes were immunoprecipitated using an anti-CDK6, anti-CDK4 or anti-PolII-antibody and analysed by PCR for the presence of p16INK4a or Vegf-A promoter sequence. One representative experiment out of three is depicted.

J) Promoter ChIP assays were performed using *Cdk6*$^{+/+}$, *Cdk6*$^{+/+}$+*Cdk6* and *Cdk6*$^{-/-}$ cells. Protein-DNA complexes were immunoprecipitated using antibodies specific for the indicated histone modification. ChIP and input DNA were analysed by qPCR for the presence of a *Vegf-A* promoter sequence (region 1 in Supplementary Figure 11c). The relative enrichment of the histone modification was determined by dividing the percentage of precipitated DNA of the *Vegf-A* promoter region (ChIP/input) by the percentage of precipitated DNA at a positive control region (ChIP/input). A *Tbp* promoter region was used as positive control for H3K9ac, H3K4me2 and H3K4me3 and a *Neurog1* promoter region was used for H3K27me3. The mean + S.E.M. of two independent experiments is shown.

K) H3K36me3 ChIP assays were performed using Cdk6$^{+/+}$ and Cdk6high cells. Protein-DNA complexes were immunoprecipitated using an antibody specific for H3K36me3. ChIP DNA was analysed by qPCR for the presence of *Vegf-A* sequences depicted in the lower panel [black rectangles; middle of the amplicon relative to the TSS (arrow symbol marked +1) of *Vegf-A*: 1 -386 bp, 2 +693 bp, 3 +2710 bp, 4 +6461 bp, 5 +8621 bp]. The relative enrichment of the histone modification was determined by dividing the percentage of precipitated DNA of the given *Vegf-A* region (ChIP/input) by the percentage of precipitated DNA of a Gapdh gene region (ChIP/input). The mean + S.E.M. of two independent experiments is shown.

L) Human *CDK6* and *VEGF-A* mRNA levels of several human B-cell lines (JVM2, SUP-B15, DOHH2, RAJI, Granta519, U931, SD1, REC1, Ramos RL-7) were analysed by qPCR. The correlation between *CDK6* and *VEGF-A* is depicted in a x,y blot.

M) Human *CDK6* and *VEGF-A* mRNA levels of several human T-cell lines (Molt13, HSB2, MKB1, PEER, LBL, HPB-ALL, CCRF, Mac2A, Jurkat, Karpas, Sudhl1, Molt4) were analysed by qPCR. The correlation between *CDK6* and *VEGF-A* is depicted in a x,y blot.

N) Human *CDK4* and *VEGF-A* mRNA levels of several human B-cell lines (JVM2, SUP-B15, DOHH2, RAJI, Granta519, U931, SD1, REC1, Ramos RL-7) were analysed by qPCR. The correlation between *CDK4* and *VEGF-A* is depicted in a x,y blot.

O) Human *CDK4* and *VEGF-A* mRNA levels of several human T-cell lines (Molt13, HSB2, MKB1, PEER, LBL, HPB-ALL, CCRF, Mac2A, Jurkat, Karpas, Sudhl1, Molt4) were analysed by qPCR. The correlation between *CDK4* and *VEGF-A* is depicted in a x,y blot.
Error bars indicate the mean ± SEM.

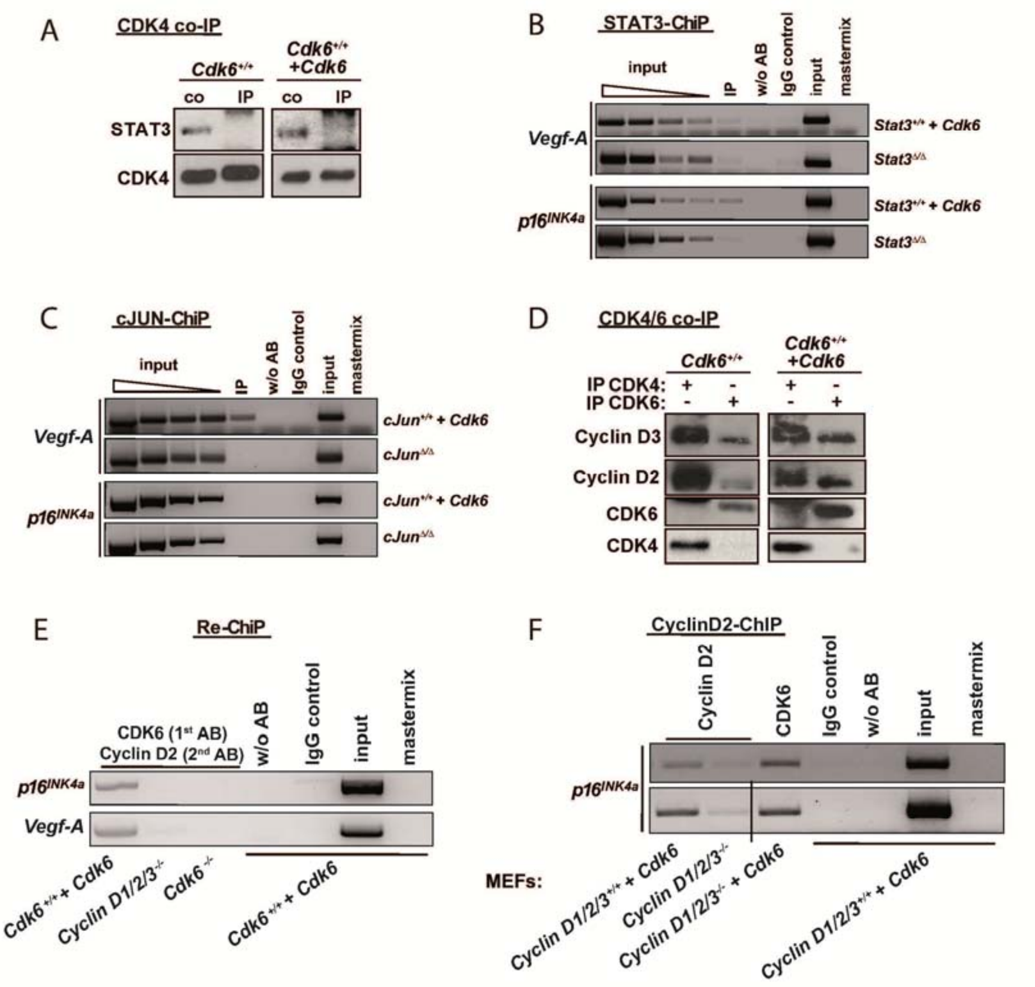

Figure S7, related to Figure 7. Interactionpartners of CDK6 to regulate transcription.

A) An anti-CDK4 co-immunoprecipitation (co-IP) was performed with *Cdk6*$^{+/+}$ and *Cdk6*$^{+/+}$+*Cdk6* cell extracts and immunoblotted for STAT3 and CDK4. Depicted is a whole lysate, as a control (co) and the CDK4 immunoprecipitate.

B, C) ChIP assays were performed using **(B)** *Stat3*$^{+/+}$+*Cdk6* and *Stat3*$^{\Delta/\Delta}$ cells. Protein-DNA complexes were immunoprecipitated using an anti-STAT3-antibody and analysed by PCR for the presence of *Vegf-A* or *p16*^{INK4a} promoter sequence **(C)** *cJun*$^{+/+}$+*Cdk6* and *cJun*$^{\Delta/\Delta}$ cells. Protein-DNA complexes were immunoprecipitated using an anti-c-JUN-antibody and analysed by PCR for the presence of *Vegf-A* or *p16*^{INK4a} promoter sequence. One representative experiment out of three is depicted.

D) An anti-CDK4 and anti-CDK6 co-immunoprecipitation (co-IP) was performed with *Cdk6*$^{+/+}$ and *Cdk6*$^{+/+}$+*Cdk6* cell extracts and immunoblotted for Cyclin D2, Cyclin D3, CDK4 and CDK6.

E) Re-ChIP assays were performed using p185$^{BCR-ABL}$-transformed *Cdk6*$^{+/+}$*+Cdk6 and Cdk6*$^{-/-}$cells as well as *CyclinD1/2/3*$^{-/-}$ MEFs. Protein-DNA complexes were immunoprecipitated using an anti-CDK6 and anti-Cyclin D2 antibody and analysed by PCR for the presence of *p16*^{INK4a} and *Vegf-A* promoter sequence. Two representative experiments out of three are

depicted.

F) ChIP assays were performed using *CyclinD1/2/3*$^{+/+}$+*Cdk6* and *CyclinD1/2/3*$^{-/-}$ MEFs. Protein-DNA complexes were immunoprecipitated using an anti-Cyclin D2 or CDK6-antibody and analysed by PCR for the presence of *p16*^{INK4a} promoter sequence. One representative experiment out of three is depicted.

SUPPLEMENTAL EXPERIMENTAL PROCEDURES

Cell culture, infection of foetal liver cells and expression vectors

Tissue culture conditions, virus preparation, infections establishment of cell-lines and colony forming assays were performed as described previously (Kollmann et al., 2011a). Expression vectors: *pMSCV-puro, pMSCV-Cdk6-puro, pMSCV-Cdk6R31C-puro* (Grossel et al., 1999), *pMSCV-Cdk6K43M-puro* (Zacharek et al., 2005), *pMSCV-Cdk4-puro, pMSCV-JunB-puro* and *pMSCV-cJun-puro.*

cJun$^{\Delta/\Delta}$ and *Stat3*$^{\Delta/\Delta}$ p185$^{BCR-ABL}$-transformed cell lines and Cyclin D1/2/3$^{-/-}$ MEFs have been described previously (Hoelbl et al., 2010; Kollmann et al., 2011a; Kozar et al., 2004).

Generation of p185$^{BCR-ABL}$-transformed Cdk6$^{-/-}$ cells with inducible expression of CDK6:

To generate cell lines with doxycycline-inducible expression of CDK6, p185$^{BCR-ABL}$-transformed *Cdk6*$^{-/-}$ cells were retrovirally transduced with pRevTet-On (Clontech) and selected with puromycin (2 μg/ml). To test for doxycycline-inducible gene expression, *Cdk6*$^{-/-}$ -tet-on cells were transduced with the pRevTRE vector (Clontech) containing a GFP cDNA and selected by growing in hygromycin (400 μg/ml). Single cell clones were generated by FACS sorting and screened for clones with the highest doxycyline-dependent induction of GFP and the lowest background expression. These *Cdk6*$^{-/-}$-tet-on cells were than transduced with the pRevTRE-tight vector (Clontech) containing CDK6 cDNA and selected by growing in hygromycin (400 μg/ml). Expression of CDK6 was induced by addition of doxycycline (0.3-10 μg/ml) (Mayerhofer et al., 2008).

Isolation of untransformed human B and T cells: Peripheral blood mononuclear cells were obtained from human peripheral blood samples over a Ficoll gradient, and B and T cells were then selected using first the EasySep FITC Selection Kit (StemCell Technologies) together with FITC-labeled antibodies to exclude all CD41$^+$, CD66b$^+$, CD56$^+$, CD36$^+$, CD16$^+$, GPA$^+$, CD71$^+$, CD33$^+$ and CD15$^+$ cells and secondary the EasySep APC Selection Kit (StemCell Technologies) together with APC-labeled antibodies to select eather CD19$^+$ B cells or CD2$^+$ T cells, according to manufacturer's instructions.

Isolation of untransformed murine B and T cells: Bone marrow of three 6 week-old C57BL/6J mice was pooled and stained with fluorescence-conjugated antibodies CD3ε and CD19. 4-way purity FACS sorting was performed using an eight-colour BD FACSAria equipped with 488, 633, 546 and 407 nm lasers

^{3}[H]-thymidine incorporation and flow cytometric analysis

^{3}[H]-thymidine incorporation and cell cycle analysis were performed as previously described (Kollmann et al., 2011a).

Colony forming assay

A defined number of p185$^{BCR\text{-}ABL}$-transformed cells were plated in growth factor-free methylcellulose (StemCell Technologies). Formed colonies were analyzed after 5 days of incubation by colony counting per dish and taking pictures. Assay was performed in duplicates.

Transplantation of tumour cells into *Rag2$^{-/-}$* and *Nu/Nu* mice

Tail vein injection and subcutaneous injection were performed as described previously (Kollmann et al., 2011a).

PD0332991 treatment:

^{3}[H]-thymidine incorporation: 1x10^{5} cells were seeded in triplets in 96-well plates and incubated with 0, 30, 100, 300, 1000, 3000 nM PD0332991. After 12h incubation, ^{3}[H]-thymidine (0,1µCi/well [MBq/well]) was added and incubated again for 12h before analyzation.

Western Blot and qPCR: 1x10^{6} cells were incubated with 0, 30, 100, 300, 1000, 3000 nM PD0332991 for 24h, harvested and analyzed .

Protein analysis and immunoblotting

Cell lysates and immunoblotting was performed as described previously (Kollmann et al., 2011a).

Co-immunoprecipitation: 1000 µg of cell lysates were incubated with 2µg of antibody on a rotating wheel at 4°C overnight, followed by 1/2 hour of incubation in the presence of equilibrated protein A sepharose beads on a rotating wheel at 4°C. After washing 4x the mixture was centrifuged and SDS loading buffer added. Heating the samples for 5 min. at 95°C separated beads and proteins. Reaction mixtures were run on a SDS polyacrylamide gel.

Treatment with bortezomib and cycloheximide: 5 x 10^{6} cells were seeded in either 10nM bortezomib, 1µM cycloheximide or a combination of these drugs. After 4h incubation immunoblot analysis was performed.

Cytoplasmic and nuclear fractionation: Fractionation was performed as described previously (Schreiber et al., 1989)..

Antibodies used in the study

**Antibodies used for histonmark analysation:**	
H3K9ac	Millipore, 07-352
H3K4me2	Millipore, 07-030
H3K4me3	Diagenode, pAb-003-050
H3K27me3	Millipore, 07-449
H3K36me3	Cell Signaling, 4909

**Antibodies used for ChIP experiments:**	
CDK6	Santa Cruz, sc-177
HA	Abcam, ab9110
CDK4	Santa Cruz, sc-260
STAT3	Cell Signalling, 9132
c-JUN	Santa Cruz, sc-1694x
PolII	Santa Cruz, sc-899
Cyclin D2	Santa Cruz, sc-593

**Antibodies used for immunoblot and co-immunoprecipitation experiments:**	
CDK6 (Fig. 2A, 2C, 2G, 4C, 7A, 7B, S1D, S1G, S2A, S2B, S2C, S3D, S4A, S4D,S5D, S5F, S11A, S11D)	SIGMA, C8343
CDK6 (Fig. 4E, 4F, S5E, S5G)	SIGMA, SAB4300596
CDK6 (immunoprecipitation)	SIGMA, C8343
α-Tubulin	Sigma Aldrich Inc., T-9026
CDK4	Santa Cruz, sc-260
STAT3	Cell Signalling, 9132
c-JUN	Santa Cruz, sc-1694x
HA	Abcam, ab9110
$p15^{INK4b}$	Santa Cruz, sc-612
$p16^{INK4a}$	Santa Cruz, sc-1207
$p18^{INK4c}$	Santa Cruz, sc-865
$p19^{INK4d}$	Santa Cruz, sc-1063
$p19^{ARF}$	Abcam Inc., Ab80
Gapdh	Cell Signalling, 2119
HSC-70	Santa Cruz, sc-7298
Cyclin D2	Santa Cruz, sc-593
Cyclin D3	Santa Cruz, sc-182
$p21^{CIP1}$	Santa Cruz, sc-6246
$p27^{KIP1}$	Santa Cruz, sc-1641

ELISA

VEGF Quantikine ELISA Kit (R&D Systems) was used in accordance with the

manufacturer´s recommendations.

RNA-isolation and qPCR analysis

RNA was isolated using TriZol (Invitrogen). First-strand cDNA-synthesis and PCR-amplification were performed using a reverse transcriptase–polymerase chain reaction (RT-PCR) kit (GeneAmp RNAPCRkit; Applied Biosystems) according to the manufacturer's instructions. qPCR was performed on an Eppendorf RealPlex cycler using RealMasterMix (Eppendorf) and SYBR Green. Each experiment was performed in triplicate and results normalized by comparison to *rplpO* mRNA expression.

Primer used in the study:

**Primer (5' - 3') used for histonmark analyzation:**		
mouse	*Cdkn2a_1*	AACACCCCTGAAAACACTGC
		TCCTGAACCCTGCATCTCTT
mouse	*Cdkn2a_2*	AGGAGTCCTGGCCCTAGAAA
		TATGCACAGGCTCTGGAATG
mouse	*Cdkn2a_3*	TTGGCAATGTGTGCAAGACT
		TCCTCCTCCTCTCCTGTTGA
mouse	*Cdkn2a_4*	CCTCAGGGATGACCTGTGTT
		GAATGCTTGCCTGGTGTTTT
mouse	*Vegfa_1*	GGCAGGGACGTATGAGGATA
		GCATGCATGTGTGTGTGTGT
mouse	*Vegfa_2*	CCAACTTCTGGGCTCTTCTC
		GCTAGCACTTCTCCCAGCTC
mouse	*Vegfa_3*	GCCACAGTGTGACCTTCAGA
		CTTTGAACCCCTTCCCAGAT
mouse	*Vegfa_4*	GGGATGAATGGTGGTGTTTC
		CTTCCCCATGTTCCCACTAA
mouse	*Vegfa_5*	CACAGCAGAGTGCAGGAGAG
		CACAGTCACCACCCAACAAG
mouse	*Tbp*	AAAGGGGAGGAGCCAGTAAG
		TGTGTAGCCCCGACTTTCTT
mouse	*Neurog1*	CAATCTTGGTGAGCTTGGTG
		GAGGCTCTGCTGCACTCC
mouse	*Gapdh*	TGAAGCAGGCATCTGAGGG
		CGAAGGTGGAAGAGTGGGAG

**Primer (5' - 3') used for qPCR analysis:**		
mouse	$p16^{INK4a}$	GTGTGCATGACGTGCGGG
		GCAGTTCGAATCTGCACCGTAG
mouse	*Vegf-A*	GCACAGCAGATGTGAATGCAG
		CGCTCTGAACAAGGCTCACA
mouse	*Cdk6*	GCTTCGTGGCTCTGAAGCGCG
		TGGTTTCTGTGGGTACGCCGG
mouse	*rplpO*	TTCATTGTGGGAGCAGAC
		CAGCAGTTTCTCCAGAGC

mouse	*pre-mRNA p16^{INK4a}*	GGGTGCTCTTTGTGTTCCGC
		GCTTTTGGACCAACTATGC
mouse	*pre-mRNA Vegf-A*	TCCCTCTACAG ATCATGCGG
		CCTGAGTGTGAAGCTCTGG
human	*Cdk6*	GGACGTGATTGGACTCCC
		AAGTATGGGTGAGACAGGG
human	*Cdk4*	GCTGACTTTTAACCCACACA
		AAAGATTGCCCTCTCAGTGT
human	*Vegf-A*	GTCGGGCCTCCGAAACCATG
		CGTGATGATTCTGCCCTCCTCCTTC
human	*rplpO*	GGCGACCTGGAAGTCCAACT
		CCATCAGCACCACAGCCTTC

Proliferation assays and analyzation of apoptosis by FACS

Cells were analyzed by a BD FACS-Canto II FACS device and BD FACS Diva software (Beckton Dickinson). Cell cycle profiles were obtained by staining 5 x 10^6 cells with propidium iodide (50 µg ml^{-1}) in hypotonic lysis solution (0.1% (w/v) sodium citrate, 0.1% (v/v) Triton X-100, 100 µg ml^{-1} RNAse) and incubated at 37 °C for 30 min before measurement via FACS. For evaluation of proliferation rate, 5 x 10^6 cells were stained with CFSE using the CellTrace CFSE Proliferation Kit (Invitrogen) and CFSE-MFI was measured over 20 h. To evaluate the onset of apoptosis, 5 x 10^6 cells were stained with propidium iodide and an APC-conjugated antibody to annexinV (BD Bioscience) and analyzed using a FACS device.

Methylation-specific PCR (MSP):

Methylation-specific PCR was performed as described previously (Kollmann et al., 2011b).

Immunohistochemistry

Tissue array technology was applied to compare samples using antibodies against CDK6 (sc-177, Santa Cruz Biotechnology), CD30 (Ber-H2, 0751, DakoCytomation), p16^{INK4a} (9511, CINtec® p16^{INK4a} Histology Kit, mtm laboratories AG), Ki-67 (Novocastra Laboratories, Newcastle, UK) and murine CD31 (Dianova, Hamburg, Germany) and using the ABC kit (Vector Laboratories) (AEC for CDK6 and DAB for p16, CD30) according to the manufacturers' recommendations. The Ki-67 signal was visualized with 3-amino-9-ethylcarbazole (ID laboratories, London, CDN) followed by a counterstaining with hematoxilin. Samples were rated positive for the individual antibodies when the staining intensity of the tumour cells was consistently higher than that of the surrounding

untransformed cells. Normal lymph nodes were used as controls.

Human CD31 immunhistochemistry was performed on whole tissue sections. CD31 (JC70A, Dako) –ABC kit: AEC according to the manufacturers recommendations.

Immunofluorescence staining for blood vessels was performed using an antibody against murine CD31 (DIA310, Dianova) and as fluorocrome Alexa Fluor ® 594 (A11007, Molecular Probes).

Image Acquisition and Protein Quantification *in vivo*

Samples were analysed with a Zeiss AxioImager Z1 microscope system with CCD camera and an automated acquisition system TissueFAXS^{TM} (TissueGnostics GmbH). The percentages of Ki-67-, CDK6-, p16^{INKa}- or CD30-positive cells were depicted as scattergrams. Pictures were digitalized, analysed and quantified. Statistical analysis was performed using HistoQuest^{TM} software (TissueGnostics GmbH, Vienna, Austria).

Vessel density of human tissue samples: Vessel density was assessed by two independent observers. CD31 immunostained sections were scanned (Aperio technologies).

Hot spots of vessel density were determined at low magnification (40x). Each CD31-stained lumen within this hot spot was counted at a magnification of 200x in an area of 0, 25 mm2 by two independent pathologists. Vessel density was prescribed as mean values of counting results.

Immunoflourescence Analysis

IF- Analyses were performed on whole tissue sections of 8 human ALCL cases. Tissue sections were deparaffinized, antigen retrieval was carried out by steamer in TE-buffer. Non specific binding sites were saturated by goatserum for 15 minutes at room temperature. The primary antibodies CDK6 (1: 50 dilution; Sigma-Aldrich, AB-13, SAB4300595) and p16^{INK4a} (9511, CINtec® p16^{INK4a} Histology Kit, mtm laboratories AG) were incubated at 4° C overnight. After washing the primary antibodies were detected with appropriate secondary antibodies 1 hour at room temperature (Alexa Flour 488 goat anti rabbit, 1: 500 for CDK6; Alexa Flour 594 goat anti mouse, 1:500 for p16^{INK4a}). After immunostaining the sections were incubated with 4’, 6-diamidino-2 phenylindole (DAPI).

Stainings for CDK6 und p16^{INK4a} were scored semiquantively as negative (-: <10% positive tumour cells, weak positive (+: 10-30% positive tumour cells or weakly positive tumour cells) and positive (++: >30% positive tumour cells).

SUPPLEMENTAL REFERENCES

Schreiber, E., Matthias, P., Muller, M. M., and Schaffner, W. (1989). Rapid detection of octamer binding proteins with 'mini-extracts', prepared from a small number of cells. Nucleic Acids Res *17*, 6419.

Hoelbl, A., Schuster, C., Kovacic, B., Zhu, B., Wickre, M., Hoelzl, M. A., Fajmann, S., Grebien, F., Warsch, W., Stengl, G., *et al.* (2010). Stat5 is indispensable for the maintenance of bcr/abl-positive leukaemia. EMBO Mol Med *2*, 98-110.

Kozar, K., Ciemerych, M. A., Rebel, V. I., Shigematsu, H., Zagozdzon, A., Sicinska, E., Geng, Y., Yu, Q., Bhattacharya, S., Bronson, R. T., *et al.* (2004). Mouse development and cell proliferation in the absence of D-cyclins. Cell *118*, 477-491.
